# Label-free measurement of antimicrobial peptide interactions with lipid vesicles and nanodiscs using microscale thermophoresis

**DOI:** 10.1038/s41598-023-39785-0

**Published:** 2023-08-03

**Authors:** Philip Rainsford, Fredrik G. Rylandsholm, Martin Jakubec, Mitchell Silk, Eric Juskewitz, Johanna U. Ericson, John-Sigurd Svendsen, Richard A. Engh, Johan Isaksson

**Affiliations:** 1 Department of Chemistry, Faculty of Science and Technology, UiT the Arctic University of Norway, 9019 Tromsø, Norway; 2 Research Group for Host Microbe Interactions, Department of Medical Biology, Faculty of Health Sciences, UiT the Arctic University of Norway, 9019 Tromsø, Norway; 3 Natural Products and Medicinal Chemistry, Department of Pharmacy, Faculty of Health Sciences, UiT the Arctic University of Norway, 9037 Tromsø, Norway

**Keywords:** Bioanalytical chemistry, Phospholipids, Analytical chemistry

## Abstract

One strategy to combat antimicrobial resistance is the discovery of new classes of antibiotics. Most antibiotics will at some point interact with the bacterial membrane to either interfere with its integrity or to cross it. Reliable and efficient tools for determining the dissociation constant for membrane binding (*K*_*D*_) and the partitioning coefficient between the aqueous- and membrane phases (*K*_*P*_) are therefore important tools for discovering and optimizing antimicrobial hits. Here we demonstrate that microscale thermophoresis (MST) can be used for label-free measurement of *K*_*D*_ by utilising the intrinsic fluorescence of tryptophan and thereby removing the need for chromophore labelling. As proof of principle, we have used the method to measure the binding of a set of small cyclic AMPs to large unilamellar vesicles (LUVs) and two types of lipid nanodiscs assembled by styrene maleic acid (SMA) and quaternary ammonium SMA (SMA-QA). The measured *K*_*D*_ values correlate well with the corresponding measurements using surface plasmon resonance (SPR), also broadly reflecting the tested AMPs’ minimal inhibition concentration (MIC) towards *S. aureus* and *E. coli. *We conclude that MST is a promising method for fast and cost-efficient detection of peptide-lipid interactions or mapping of sample conditions in preparation for more advanced studies that rely on expensive sample preparation, labelling and/or instrument time.

## Introduction

Antimicrobial peptides (AMPs) have attracted increasing attention as a source of inspiration to combat the looming antimicrobial resistance crisis as the discovery of new antibiotics classes has ground to a halt^1^. AMPs are a class of peptides that have antimicrobial activity, though they are also known to possess some anti-fungal and anti-cancer properties^2,3^. They are typically short, cationic peptides of about 12–50 amino acids, with typically at least 4 residues required for activity^4^. Found in most living organisms, they are natural and indispensable components of innate immune defences^5–8^. Currently, more than 20 000 peptide sequences with antimicrobial properties are published in dedicated depositories^9–13^, providing a pool of potential therapeutic candidates. The antimicrobial mode of action of most AMPs seems to be to target the integrity and/or the electric potential of the membrane bilayer, or to have multiple targets and combinations of modes of action—something that makes resistance more difficult to develop and comes with a higher fitness cost to maintain. With some exceptions, this contrasts with traditional antibiotics, which usually have a well-defined target. A second advantage of AMPs is that there is no requirement to entirely cross the membrane, with membrane active peptides often exerting their activities by disrupting, permeabilising or lysing the bacterial membrane itself^14,15^.

AMP affinity towards bacterial membranes is often described in two ways: As a binding event which can be described via the dissociation constant *K*_*D*_^16^; or as a biphasic system, where the AMP interaction with lipids is viewed as a partitioning between a lipid phase and an aqueous phase, characterized by the partitioning constant *K*_*P*_^17^. Both *K*_*D*_ and *K*_*P*_ are thus useful descriptors for screening compounds based on their interactions with the target bacterial membrane.

Current methods used to assess lipid affinity typically suffer from several disadvantages. They may not be applicable to stronger bindings (NMR)^18^, require labelling that impacts binding (fluorescence assays)^19^, be time-consuming (NMR and SPR)^20,21^, demand large quantities of samples (NMR) or require fine-tuning for each individual compound (SPR and ITC)^20,21^. Microscale thermophoresis (MST) is a method that does not possess these disadvantages^22^. It is a simple but powerful tool, that enables binding parameter determination by observation of the effects of binding on a fluorophore and on the relative thermophoretic properties of the complexes formed. Bindings are obtained by monitoring changes in the fluorescence intensity of a complex over time upon exposure to an IR laser. The laser heats a series of samples with differing ligand concentrations, causing a redistribution of molecules according to their relative Gibbs energies in solution at the different temperatures (thermophoresis). Changes in fluorescence due to ligand binding effects are monitored upon heating and re-cooling. The method is sensitive to changes in fold, shape, solvation shell, charge, or overall size of the ligand-bound complex. These changes can affect the local environment of a fluorophore by altering dynamic and static quenching, as well as the thermophoretic properties of the complex, and make MST sensitive to potentially minor changes in properties that arise from binding^23^. MST is currently primarily used to assess biomolecular interactions, including the binding of ligands to various substrates^22^ and polymerisation^24^. A previous related application of MST by Yu et al. assessed the binding of an AMP using FITC-labelling^25^. MST can be performed using fluorophore labelling or label-free, taking advantage of intrinsically fluorescent amino acids like W, Y and F. The near ubiquity of W in many AMPs provides an opportunity to study AMP binding properties directly by utilising the intrinsic fluorescence of W. Additional important advantages of MST are low sample requirements, short measurement times and simple experimental setup^20,21,26^.

**Figure 1:**
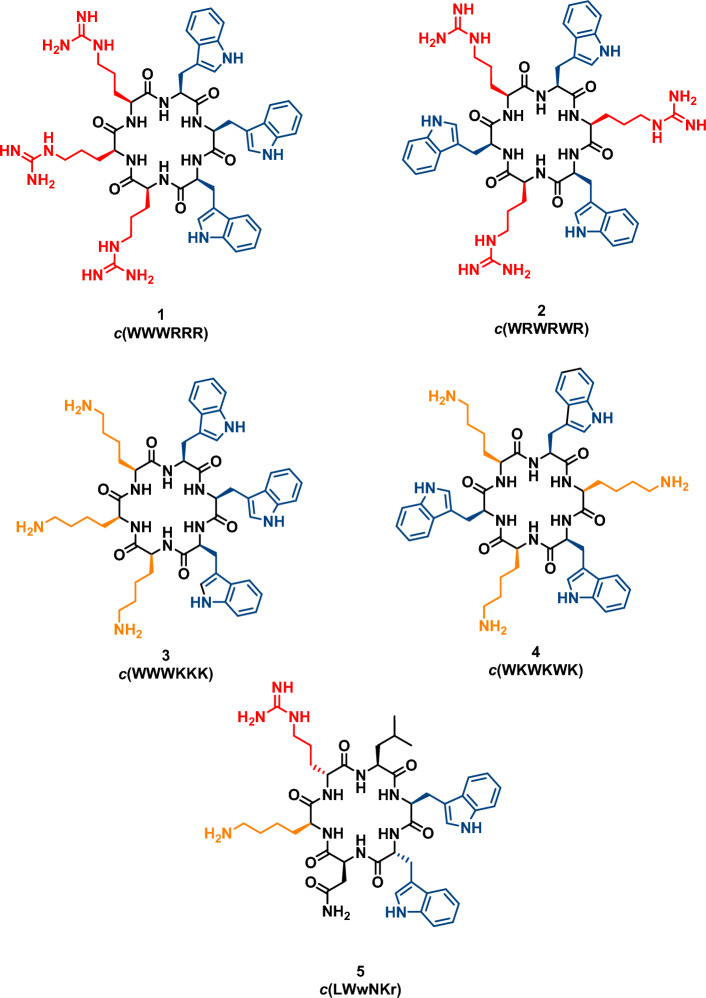
Structures of the five AMPs Coloured red/orange—R/K, blue—W. Uppercase letters indicate l-amino acids, lowercase letters indicate d-amino acids.

Herein we demonstrate that MST is a viable method for the rapid label-free determination of AMP affinity towards lipid bilayers. We used label-free MST to study five cyclic antimicrobial hexapeptides (Fig. 1) and their bindings to three lipid systems, comparing the results to SPR data on binding to lipid vesicles.

AMPs **1**–**4** were selected based on a previously established pharmacophore of alternating versus clustered distribution of charged and hydrophobic moieties^27–31^, where the clustering of W and charged residues is shown to be positively correlated with antimicrobial activity^4^. AMPs **1–4** were confirmed to have antimicrobial activity (Table 1) and are a combination of alternating and clustered W residues with either R or K residues. AMP **5** was included as a negative control, as it was inactive against the tested bacterial strains (Table 1) and it showed poor lipophilic preference.

**Table 1:** Summary of the cyclic hexapeptides and their minimal inhibition concentration (MIC).

#	Sequence	*E. coli* MIC (µg/mL) (ATCC 25922)	*S. aureus* MIC (µg/mL) (ATCC 9144)	Net charge	Hydrophobic residues
1	WWWRRR	8	4	+ 3	3
2	WRWRWR	32	32	+ 3	3
3	WWWKKK	8	32	+ 3	3
4	WKWKWK	64	128	+ 3	3
5	LWwNKr	> 256	> 256	+ 2	2

The three different lipid systems that were selected for the study were large unilamellar vesicles (LUVs), styrene-maleic acid (SMA) nanodiscs^32^, and styrene-maleic acid functionalised with quaternary ammonium (SMA-QA) nanodiscs^33^. The different systems were chosen to investigate both the suitability of different lipid models to the application of MST and lipid-AMP binding, and the impact the choice of model system can have on the binding. SMA nanodiscs and vesicles were chosen as they have been previously utilised in MST^23,34^, and the effects of differences in surface curvature could be investigated. SMA-QA was selected as it possesses a cationic polymer belt, in contrast to SMA which has an anionic belt.

As the electrostatic attraction of cationic AMPs and anionic lipids is known to enhance their interactions, the association of the AMPs with both pure DMPC and a mixture of 95% DMPC and 5% PG were assessed in each of the three lipid systems. Finally, SPR was used as an orthogonal method to which the MST derived bindings could be compared.

## Results and discussion

### Surface plasmon resonance (SPR) characterization

**Figure 2:**
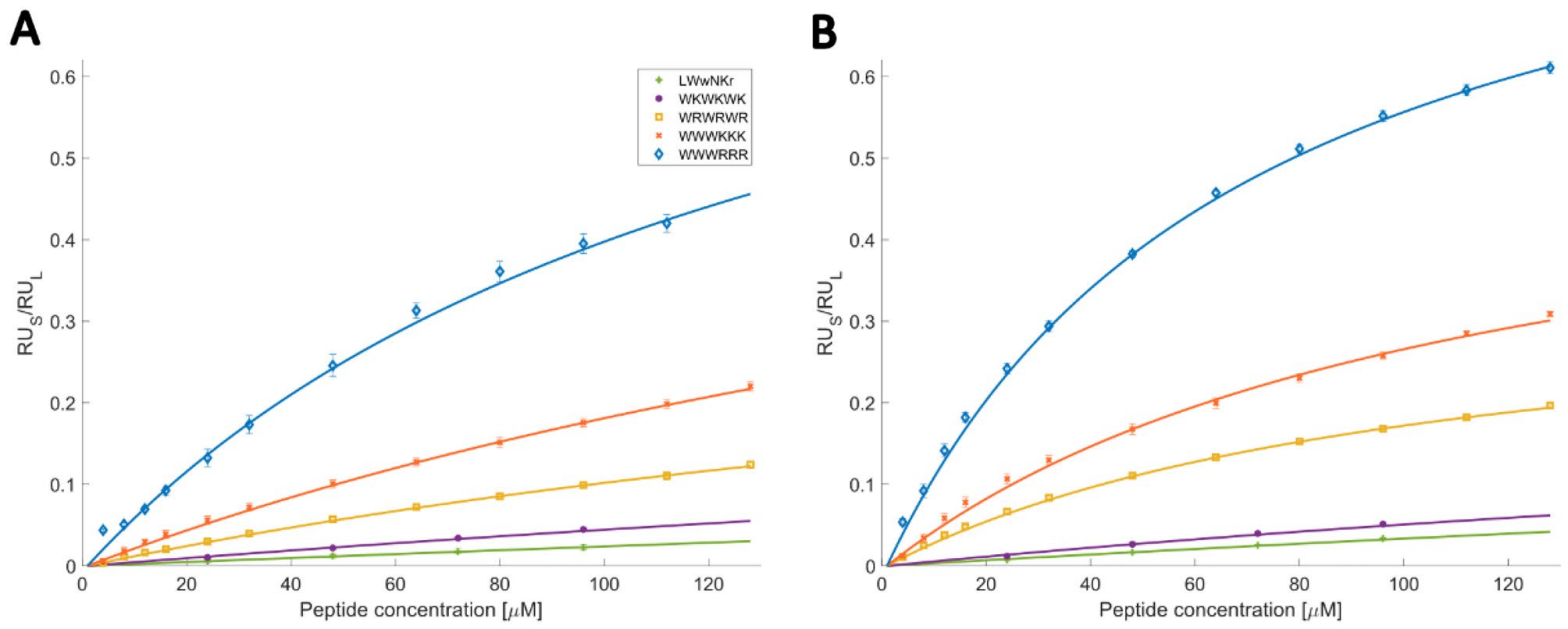
Ratio of RU_s_ (peptide signal) and RU_L_ (lipid bilayer) obtained from steady state using SPR for (**A**) 100% DMPC LUVs. (**B**) 95% DMPC 5% DMPG LUVs. Scatter plot present experimental values and full line represent fit obtained from Eq. (2). Full sensograms are available at the UiT open research data depository^35^.

The interactions between AMPs **1**–**5** and LUVs were first investigated by SPR as a benchmark for the MST measurements. The data was acquired using the same lipid compositions as used in the MST data acquisition. LUVs were immobilized on an L1 chip, and an increasing concentration of AMPs was injected over them. Figure 2 displays typical steady state fits of the SPR measurements, and the disassociation- and partitioning constants, *K*_*D*_ and *K*_*P*_, extracted after 180 s are listed in Tables 3 and 4. The dissociation rate, *k*_*off*_, was calculated from the dissociation step, using the methodology presented by Figueira et al*.*^21^, summarized in Table 2.

**Table 2:** Summary of k_off_ of AMPs **1**–**5** evaluated by SPR.

#	Peptide	*k*_*off*_ PC (s^−1^)	*k*_*off*_ PC/PG (s^−1^)
1	WWWRRR	0.22 ± 0.02	0.19 ± 0.01
2	WRWRWR	0.87 ± 0.19	0.48 ± 0.05
3	WWWKKK	0.48 ± 0.07	0.32 ± 0.05
4	WKWKWK	0.90 ± 0.24	1.32 ± 0.05
5	LWwNKr	1.76 ± 0.12	1.75 ± 0.16

SPR showed values for *K*_*D*_, *K*_*P*_ and *k*_*off*_ in the range of 50–800 μM, 1–150 10^−3^ and 0.2–1.0 s^−1^ respectively. **1** was the strongest binding compound, followed by **3, 2** and **4,** with inactive AMP **5** being the weakest. This trend was also preserved when anionic lipids were present, but as expected, the overall affinities of all compounds was increased. The *k*_*off*_ values also followed this trend with the most active AMP, **1**, having the slowest dissociation. The overall conclusion from SPR points towards increased affinities of the clustered peptides over alternating ones towards both zwitterionic and ionic lipid bilayers.

### Vesicle MST response profiles

**Figure 3:**
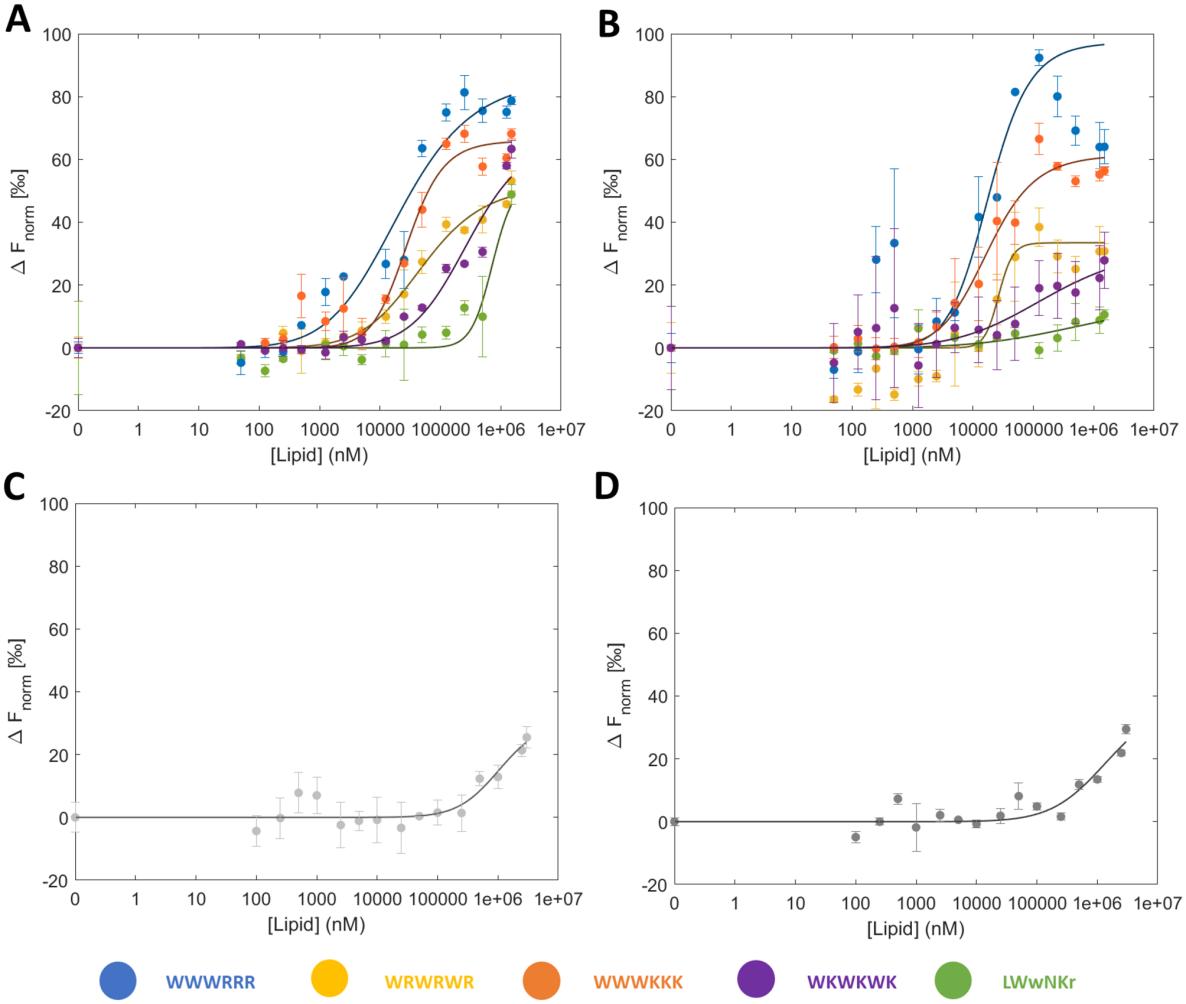
MST response against lipid concentration and average K_D_ fits of **1**–**5** in different lipid vesicles compositions. The upper panels show 100 nm vesicles. (**A**) 100% DMPC. (**B**) 95% DMPC 5% DMPG. The lower panels show the blank MST response. (**C**) 100% DMPC. (**D**) 95% DMPC/5% DMPG. Error bars represent the standard deviation of the triplicates.

For K_D_ evaluation by MST, F_Hot_ was registered during the temperature related intensity change (TRIC) phase of the MST trace (see “Methods”). The advantage of evaluating *K*_*D*_ during the TRIC phase is that any potential effects of prolonged heating on the thermostability of the sample that may influence the binding can be avoided^36^. The dose–response profile of MST response against lipid concentration for **1**–**5** largely follows a sigmoidal curve shape (Fig. 3A,B). In the cases of **5** and **4**, the weaker binding resulted in truncation of the sigmoidal curve, with the asymptotic region at high lipid concentration not being fully sampled.

A blank series consisting of only lipids was collected for each lipid composition, and a weak MST response could be detected at the highest lipid concentrations despite the lack of a fluorophore. The blank response was stable up to concentrations around 1 mM. The two to three highest lipid concentrations measured did however build up a background response above the noise level of the baseline (Fig. 3C,D), most likely due to increasing turbidity. The added unspecific response to the last two data points thus imposes a limitation on how weak interactions can be reliably measured (mM lipid concentration range). The turbidity did however not notably impact the extraction of F_norm_ in the presence of AMPs (carrying the fluorophore), as the much stronger signal of the fluorophore did not display any signs of inheriting the turbidity contribution to F_norm_ from the blank profile (raw data shown in Fig. S1). This suggests that W fluorescence was the dominant contribution to the measured response when it was present. It is however not possible to rule out that the light scattering effect may dominate the response for very weak binders. Peptide **5**, for example, shows a change in F_norm_ for the final two points together with DMPC liposomes, the same points that are significantly affected by light scattering effects in the blank measurement (Fig. 3A). The effect is however absent for the same peptide together with DMPC:PG liposomes even though the two blanks behave similarly, demonstrating that potential light scattering contributions are difficult to predict or compensate for.

That light scattering is present at higher lipid concentrations may raise concerns that the density of lipids could potentially hinder the thermophoresis of analytes in the sample, even for non-interacting species. However, as demonstrated by Yu et al.^25^, the MST response of non-interacting moieties, demonstrated with FITC, is unaffected up to lipid concentrations of 2.5 mM. Further, sampling of the MST response in the TRIC phase of the MST trace means that thermophoretic effects are less dominant, minimising the risk of artefacts caused by the lipids^37^.

### Nanodisc MST response profiles

**Figure 4:**
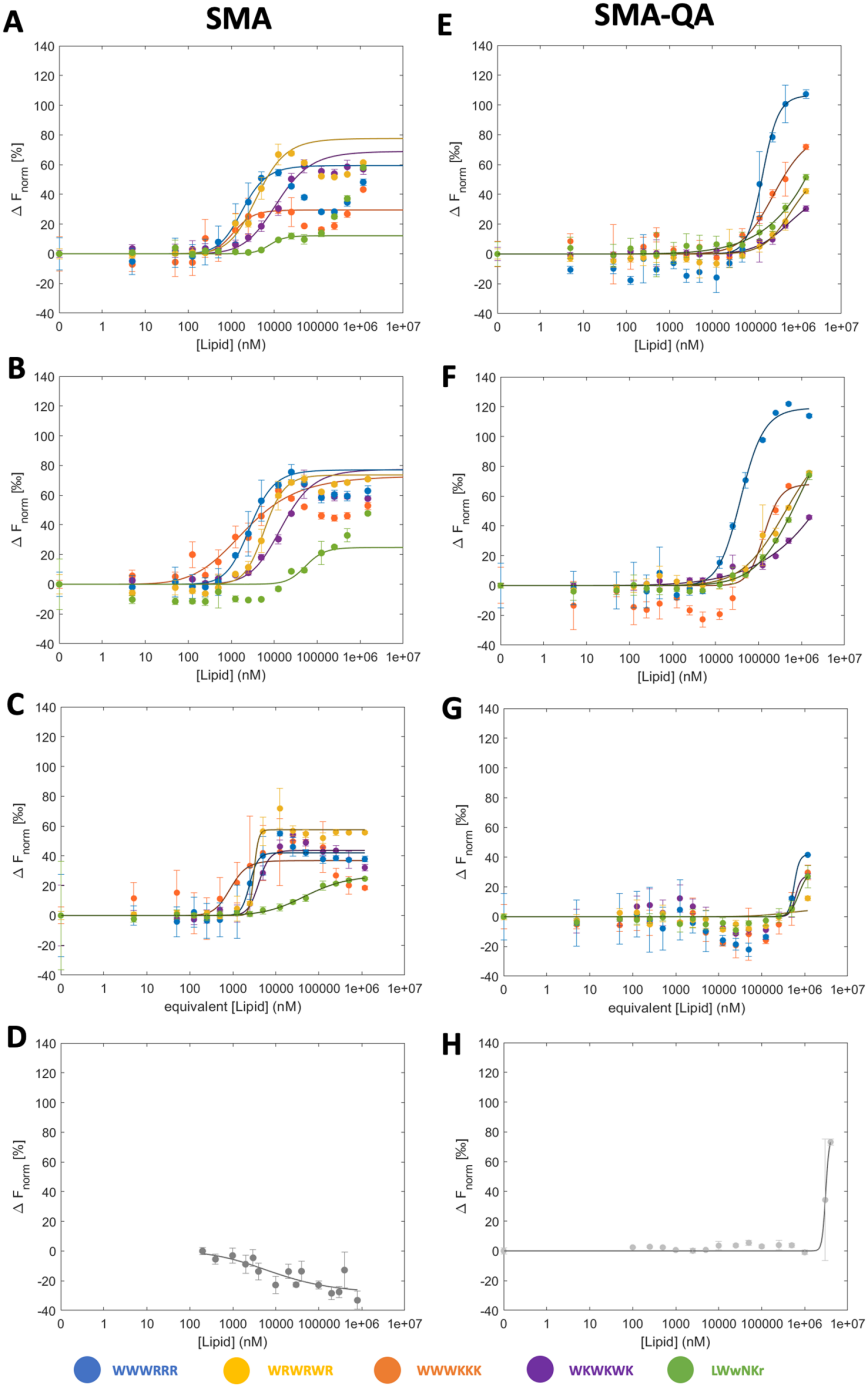
MST response against lipid concentration and K_D_ fits of **1**–**5** in different lipid nanodisc compositions. The left panels show SMA nanodiscs, and the right SMA-QA nanodiscs consisting of (**A**/**E**) 100% DMPC, (**B**/**F**) 95% DMPC/5% DMPG, (**C**/**G**) polymer (no lipids*), and (**D**/**H**) nanodisc only (no peptide). *Equivalent [lipid] is used to estimate the amount of SMA that would be present at that lipid concentration in the corresponding nanodiscs. Error bars represent the range in the triplicates.

The MST data for two nanodisc types, SMA and SMA-QA, were evaluated using the same methodology as the vesicle data, with F_Hot_ taken during the TRIC, and F_norm_ plotted against log[lipid] and fit to Eq. (6). The binding profiles of AMPs binding to SMA and SMA-QA nanodiscs show significantly different behaviours (Fig. 4).

The SMA-QA nanodiscs produce a sigmoidal-like response curve (Fig. 4D,E), as was observed for the vesicles, though the curve is right-shifted to higher lipid concentrations, i.e., AMPs bind more weakly to the SMA-QA nanodiscs than to the corresponding vesicles. The consequence of the right-shift is that the binding curves of the weaker binders are not fully sampled, and in some cases only a minimum K_D_ can be determined. As with vesicles, at the highest lipid concentrations light scattering can become a factor in the measurement of F_norm_. The result is that it becomes impractical to fully sample the binding curve by further increasing the lipid concentration, as an increased influence of light scattering effects would be expected.

By contrast, the SMA-nanodiscs response is left-shifted to lower lipid concentrations, corresponding to a stronger binding than to the corresponding vesicles (Fig. 4A,B). The curves also deviate from the sigmoidal F_norm_ profile with a secondary drop in F_norm_ after reaching the maximum, in the mM range. Such profiles have previously been observed in the MST response of higher stoichiometric bindings where additional interacting ligands gave rise to a new species of the complex^22^. In the case of nanodisc-AMP interactions, the most plausible explanation is that direct interactions between **1**–**5** and the SMA polymer gives rise to the atypical binding profile. A direct interaction between the free SMA polymer and the AMPs can indeed be observed in the high nM–low µM range in a control experiment without lipids present (Fig. 4C,G). The presence of strong interactions to the SMA polymer, and a multiple-phase response profile, indicates that the interaction with the polymer dominates the measurement to the extent that the AMP-lipid interaction cannot be directly measured in SMA nanodiscs (Table 3). The strength of the interaction with the SMA polymer, together with its role in stabilizing the nanodisc, led to the assumption that the system would be significantly perturbed. Therefore, no further attempts were made to deconvolute multiple interactions from these response curves.

**Table 3:** Summary of K_D_ determined using SPR and MST. Errors represent the standard deviation of the triplicates. Where K_D_ is preceded by ‘ > , the value represents a minimum value due to insufficient curve sampling and no error is reported.

Peptide	SPR (K_D_ μM)		Vesicle (K_D_ μM)		SMA-QA (K_D_ μM)		SMA (K_D_ μM)	
	DMPC	DMPC/PG	DMPC	DMPC/PG	DMPC	DMPC/PG	DMPC	DMPC/PG
LWwNKr	2548 ± 493	1033 ± 58	> 670	> 650	–	> 1000	–	–
WKWKWK	712 ± 27	474 ± 45	282 ± 58	112 ± 29	> 713	–	8.6 ± 0.5	9.8 ± 0.3
WRWRWR	318 ± 62	105 ± 7	73 ± 53	24 ± 7	> 541	> 615	2.7 ± 0.5	5.6 ± 1.1
WWWKKK	302 ± 32	112 ± 15	28 ± 3	17 ± 13	267 ± 47	145 ± 55	1.1 ± 0.3	1.1 ± 0.5
WWWRRR	142 ± 35	70 ± 1	21 ± 3	10 ± 5	142 ± 17	40 ± 5	1.2 ± 0.5	2.2 ± 0.4

The strong interaction to the SMA polymer can be attributed to the rich anionic maleic acid content (deprotonated at pH 7.4), resulting in favourable electrostatic interactions to the cationic AMPs. Consequently, no such binding is observed for the SMA-QA polymer, which is instead rich in cationic moieties, and thus has the attractive electrostatic interaction potential to cationic AMPs replaced by a repulsive potential, which is reflected in the response profiles being shifted towards weaker binding.

### K_D_ comparison

**Figure 5:**
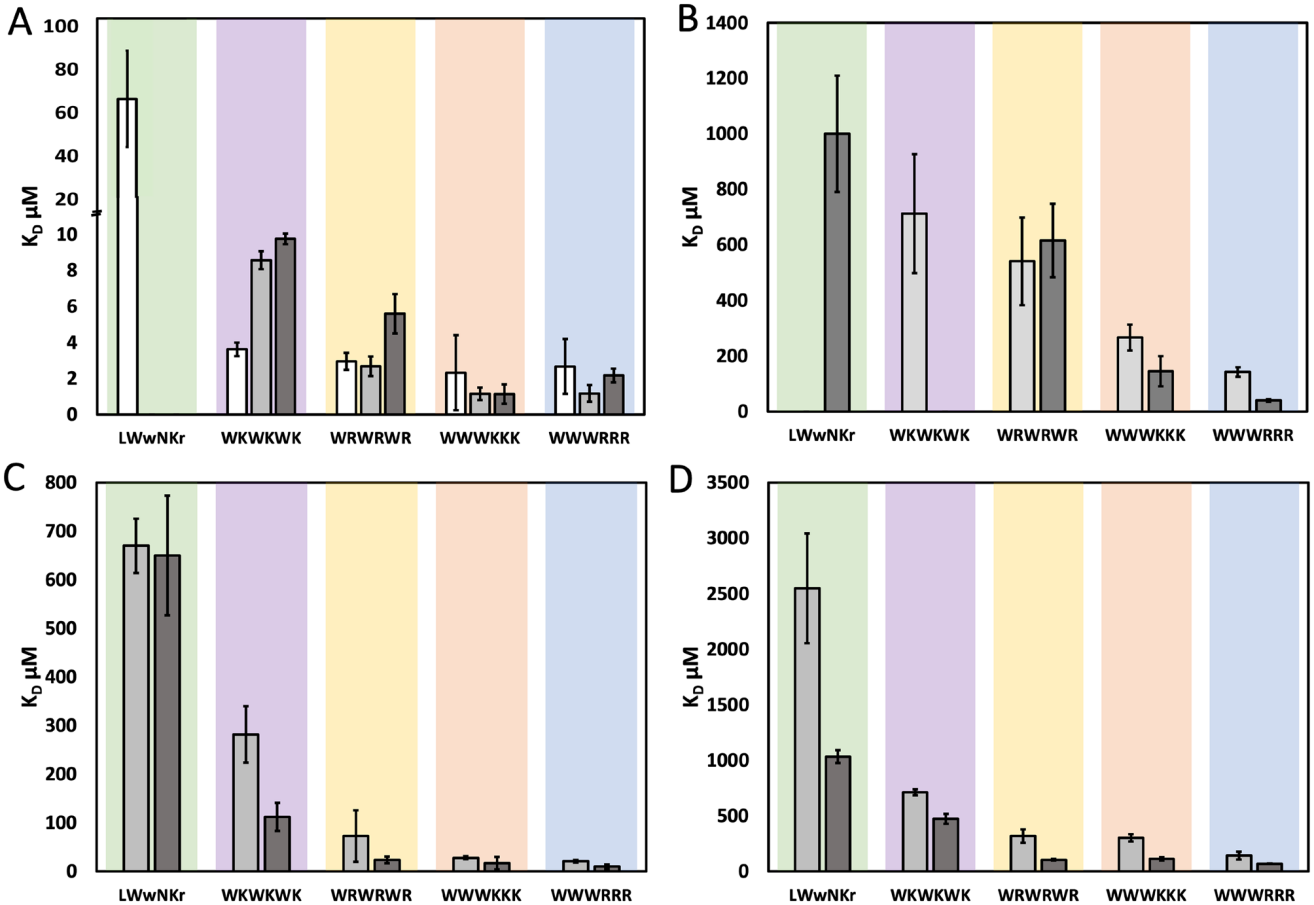
K_D_s determined by MST and SPR to DMPC (light grey) and DMPC/PG (dark grey). (**A**) K_D_ determined using SMA nanodiscs (white: SMA polymer), (**B**) K_D_ determined using SMA-QA nanodiscs, (**C**) K_D_ determined using 100 nm vesicles, (**D**) K_D_ determined using SPR and Vesicles (extruded through 100 nm filter). Error bars represent the standard deviation of the triplicates.

Comparison of the MST and SPR derived *K*_*D*_ for LUVs shows that the absolute *K*_*D*_ obtained are systematically offset by an approximate factor 4 (Fig. 5C,D and Table 3), but the relative values within each dataset result in the same stratification of the peptides binding strength to LUVs as measured by MST and SPR. The inactive peptide **5** had a considerably higher *K*_*D*_ compared to **1**–**4**, though the profile of **5** could not be fully sampled. Therefore, only the minimum *K*_*D*_ was determined, providing an explanation as to why MST did not identify the same strong dependence on the lipid composition as SPR for this peptide. Consistent with SPR, the clustered sequence peptides **1** and **3** had significantly stronger binding than **4**. This is also consistent with the determined MICs, with **1** and **3** being the most active peptides. These observations are in line with previous reports that clustering of W residues is positively correlated with antimicrobial activity^4^.

The SMA-QA derived *K*_*D*_ compare favourably with the SPR results in the instances where the curve is adequately sampled (Fig. 5B, Table 3). However, as the *K*_*D*_ approach the mM region, the SMA-QA data set are not sampled sufficiently to produce reliable results. This is expressed most evidently as large errors, where, with no maximum reached in the sampled lipid concentrations, the fitted K_D_ is sensitive to outliers and small deviations in the slope of the curve. One example of insufficient sampling and the resulting error is shown by the poor reproduction of the PC/PG discrimination observed in SPR for **2**. Importantly though, when the curve is adequately sampled, the K_D_ error is consistent with user reported MST errors^26^.

This is however not the case for the more active AMPs where the binding strength is well inside the sampling range. SMA-QA nanodiscs are thus only viable for assessing AMP-lipid interactions stronger than the mM range by MST.

The SMA discs exhibit overestimated binding strengths, showing all peptides to have apparent *K*_*D*_ of 10 µM or lower (Table 3), and show poor discrimination between PC and PC/PG lipids (Fig. 5A). The lack of discrimination is attributed to the strong interaction with the anionic SMA polymer dominating the response and masking the expected binding enhancement from the addition of the anionic lipids.

In general, the presence of PG lipids leads to a decrease in *K*_*D*_ for all peptides by an approximate factor of 2 when the sigmoidal binding curve could be fully sampled. In previously published work, Christiaens et al.^38^ found that individual peptides had a broad range of binding strengths, from a weak 350 µM towards PC vesicles, to stronger binding in the low µM-nM range to vesicles rich in anionic charges. Analysing MSP-nanodiscs by ITC and NMR, Zhang et al.^39^ observed binding to anionic lipid nanodiscs in the range of 1–2 µM. The *K*_*D*_s measured by MST are thus in line with results in the literature that shows that AMPs can bind in the low µM range to both vesicles and nanodiscs in the presence of anionic lipids, with an expected weakening of the interaction towards zwitterionic membranes. It is known that cationic AMPs have a selectivity towards bacterial membranes where anionic lipids and LPS are present on the outer membrane over cells with a more neutral surface. While the reduction in *K*_*D*_ upon introduction of anionic lipids observed for **1**–**5** is not as large as described in the above studies, the anionic component introduced in this work (5%) is low in comparison to the 20% used by Zhang et al. and Christiaens et al.

The calculated *K*_*D*_ in this work is fit using a two-state model^40^. This is however not necessarily expected to be an accurate representation of lipid interactions, where the target has no defined binding site, and it is possible that self-aggregation on the lipid surface, saturation effects, cooperative- or competitive binding will occur that will influence the binding of further AMPs^41,42^. For this reason, *K*_*D*_ is a useful illustrative and communicable descriptor of AMP-lipid binding, but care would be advised to not overinterpret the absolute value. The measured apparent K_D_ is best considered a composite value representing multiple processes of a complex interaction, that is system- and method dependent—as also highlighted in this work.

### Fluorescence intensity and K_P_

The partition coefficient (*K*_*P*_) describes the preference of compounds for lipid or aqueous phases, with a high *K*_*P*_ indicating a greater preference for the lipid phase. *K*_*D*_ in contrast describes the bound state as a molecular complex rather than phase. Both *K*_*D*_ and *K*_*P*_ are useful descriptors of AMP-lipid interactions, and instruments that can determine both can offer users a great deal of flexibility in exploring biophysical interactions. To explore the possibility to determine the *K*_*P*_ of AMPs **1**–**5** using MST, the single wavelength fluorescence intensities measured by the MST instrument were used. This data is routinely acquired during the MST measurements during the initial phase of the MST trace for use as the *F*_*Cold*_ measurement and is reported as the ‘initial fluorescence’. Thus, no additional acquisition time is required. To the best of our knowledge, this is the first attempt to make use of this data to determine *K*_*P*_ using MST hardware.* K*_*P*_ was extracted by fitting the data to a hyperbolic partition curve described by Eq. (8), after removal of non-hyperbolic points^43^.

**Figure 6:**
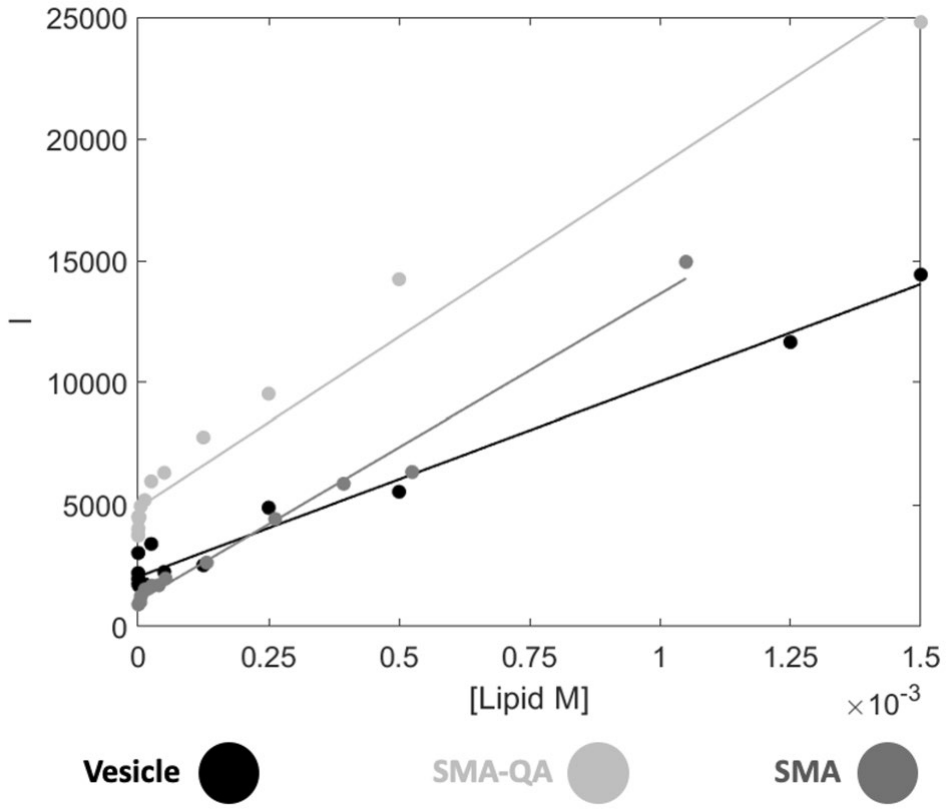
The measured fluorescent intensities of the blank lipid only samples of SMA and SMA-QA nanodiscs, and vesicles.

Lipid-only blanks were collected to assess the effect the turbidity had on the measurements (Fig. 6). For the vesicles, this is a modest signal that increases linearly in the mM lipid concentration range. The increase is consistent with the observed MST response reported above (Fig. 3C,D), attributed to light scattering effects. Both types of SMA nanodiscs similarly follow a mostly linear trend but the signals are more intense (Fig. 6).

**Figure 7:**
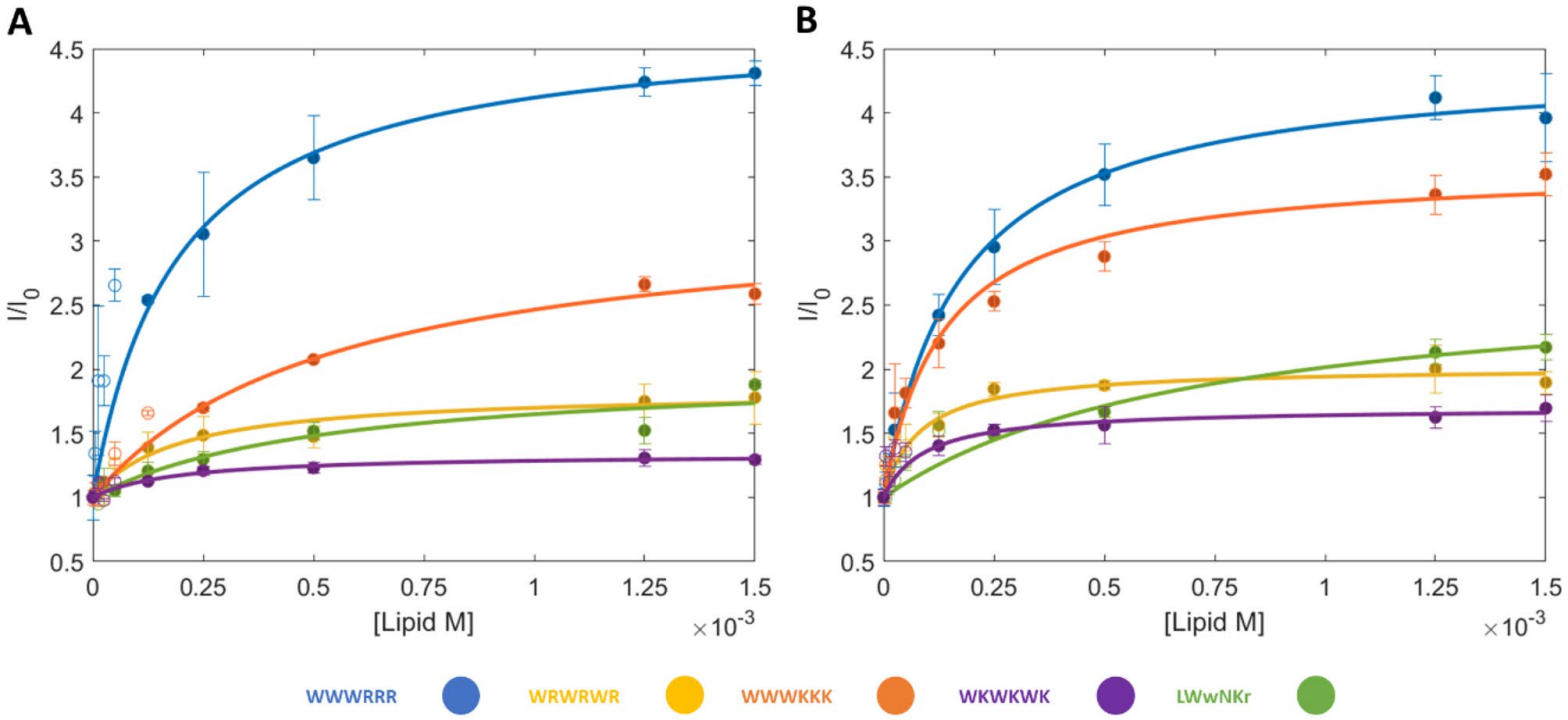
Initial fluorescence and K_P_ fits of **1**–**5** in different lipid compositions of LUVs. (**A**) 100% DMPC. (**B**) 95% DMPC with 5% DMPG. Lines and filled circles indicate the fit and the points used, unfilled circles are points not included. Error bars represent the standard deviation of the triplicates.

The background fluorescence for the vesicles is modest at the initial concentrations compared to the measured fluorescence intensities of the AMP and does not have the same profile as lipid concentration is increased (Fig. S2). A larger increase is observed for the highest lipid concentrations, showing the influence of the turbidity of the system (Fig. 6). The SMA-QA nanodiscs initial background fluorescence signal is significantly stronger, and a substantial level is maintained over the measured concentration range. As with the vesicles and SMA-nanodiscs, the intensity profile of the SMA-QA sample with AMP present does not match the profile when the AMP is present (Fig. S2). For this reason, it is not possible to subtract the blank baseline from the AMP signal. Hence, the final points carry increased uncertainty because of potential, but inconsistent, light scattering contributions (Fig. 7 and Fig. S3).

Many AMPs, especially when using vesicles, showed a spike in fluorescence at low lipid concentrations (at high peptide:lipid ratio). This phenomenon has previously been described by Melo and Castanho^44^, where they attribute the deviation to the saturation of the bilayer with AMP that prevents the uptake of additional AMPs. It has also been attributed to changes in conformation and peptide-peptide interactions within the bilayer when it is saturated with AMPs^45^. To extract *K*_*P*_ where deviations are observed, the deviant points are excluded from the fit. At lower lipid concentrations (high peptide:lipid ratio), a critical point is reached where the hyperbolic model is no longer followed and points beyond the critical point cannot be described by Eq. (8). It should be noted that the removal of points introduces uncertainty and increases the error of the extraction of *K*_*P*_; this is particularly problematic as the hyperbolic shape is best described by the initial points along the curve, but these are also the points that are the most affected by the high peptide:lipid ratios^43,44^. For both SMA-QA and LUVs, many points deviated from the hyperbolic shape, and a large number of points needed to be removed, typically leaving the final 4–5 points for the final fit.

**Figure 8:**
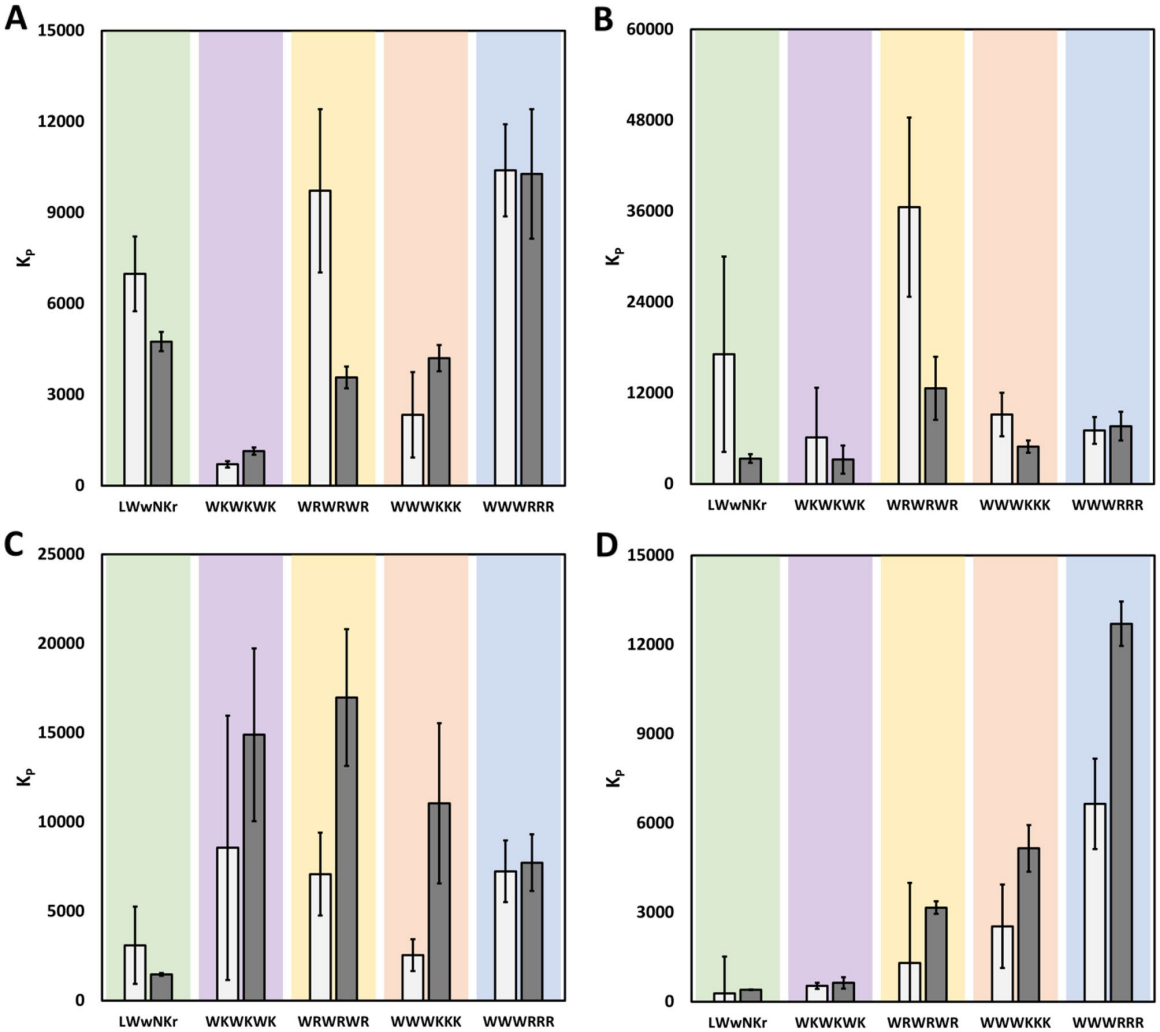
K_P_s determined by MST with DMPC (light grey) and DMPC/PG (dark grey) compared to SPR. **A** K_P_ determined using MST and SMA-nanodiscs. **B** K_P_ determined using MST and SMA-QA-nanodiscs. **C** K_P_ determined using MST and 100 nm vesicles. **D** K_P_ determined using SPR and 100 nm vesicles. Error bars correspond to the range of values for the triplicate fits.

In brief, the SPR determined *K*_*P*_ follows the *K*_*D*_ trend that **1** > **3** > **2** > **4** > **5** for both lipid compositions, with *K*_*P*_ determined in the range of 0.3–7 × 10^3^ for DMPC and 0.4–13 × 10^3^ for DMPC/PG. The MST derived *K*_*P*_ on the other hand are inconsistent with both the SPR results and the MST derived *K*_*D*_, including the expected differences in partitioning to DMPC and DMPC/PG (Table 4 and Fig. 8). The difficulties in the MST *K*_*P*_ extraction compared to SPR likely lies in the intrinsic differences in the methods. Label-free MST relies on the intrinsic fluorescence of W and the instrument measures the intensity at a fixed wavelength. The fluorescent intensity of W is influenced by static and dynamic quenching and may experience blue-shifting, processes that differ significantly between different environments, modes of binding and tendency to self-aggregate^46^. Significant blue-shifts of the W emission will displace the signal maximum to varying degrees away from the static detection frequency, resulting to a lower signal intensity being detected. Thus, there are additional factors that can negatively affect the detected signal in addition to the phase distribution.

**Table 4:** Summary of K_P_ determined using SPR and MST K_P_ × 10^3^. Bold: good correlation with SPR. Italics: reasonable correlation. Errors correspond to the standard deviation of the triplicate fits.

Peptide	SPR K_P_ × 10^3^		Vesicle K_P_ × 10^3^		SMA K_P_ × 10^3^		SMA-QA K_P_ × 10^3^	
	DMPC	DMPC/PG	DMPC	DMPC/PG	DMPC	DMPC/PG	DMPC	DMPC/PG
LWwNKr	0.28 ± 0.01	0.40 ± 0.02	3.10 ± 2.17	1.47 ± 0.07	6.98 ± 1.24	4.75 ± 0.32	17.12 ± 12.90	3.35 ± 0.57
WKWKWK	0.53 ± 0.01	0.63 ± 0.03	8.56 ± 7.41	14.90 ± 4.84	**0.70 ± 0.11**	*1.14* ± *0.12*	6.15 ± 6.54	3.23 ± 1.87
WRWRWR	1.30 ± 0.09	3.16 ± 0.15	7.08 ± 2.32	16.97 ± 3.83	9.72 ± 2.69	**3.57 ± 0.36**	36.55 ± 11.82	12.62 ± 4.16
WWWKKK	2.53 ± 0.08	5.16 ± 0.34	**2.55 ± 0.89**	11.05 ± 4.49	**2.34 ± 1.40**	**4.21 ± 0.43**	9.16 ± 2.86	**4.93 ± 0.81**
WWWRRR	6.65 ± 0.80	12.71 ± 0.16	**7.24 ± 1.73**	7.73 ± 1.58	10.40 ± 1.52	**10.27 ± 2.13**	**7.07 ± 1.78**	7.63 ± 1.89

Together, these results showed that the explored MST method was unreliable to extract *K*_*P*_ for our panel of AMPs, and that care should be taken in the interpretation of the obtained results. However, there are some correlations present that suggests that with some further work, particularly around alleviating some issues around the blue-shifting, that MST might become a viable tool to estimate *K*_*P*_ in the future. The spectral shift technology employed in newer Nanotemper devices may be ideal to further explore *K*_*P*_ extraction by MST^47^.

### Vesicle and nanodisc comparison

The SMA-QA- and vesicle derived *K*_*D*_ showed differences between the two lipid systems. The vesicles produced had a diameter of ~ 140 nm and should therefore consist of approximately 200,000 lipids (with a molecular weight of ~ 140 MDa). In comparison the 22 nm DMPG SMA-QA nanodiscs contain approximately 1300 lipids (lipid weight of ~ 760 kDa), but also a substantial fraction of polymer (Table 5). The AMPs used have molecular weights between 884 and 1027 Da, therefore when multiple AMPs are binding to a single disc, the relative change in weight, size, and shape of the nanodisc-complex will be different than with a vesicle-complex, such relative changes may impact the F_norm_ measurement as MST is sensitive to these properties of the complexes measured^48^. Furthermore, the fractions of the lipids in the models that are accessible by the AMPs differ. In nanodiscs both sides of the bilayer are accessible, and potentially enable cooperative interactions from opposite sides of the discs. In contrast, for vesicles only the outer leaflet of the vesicle surface is initially accessible, with the inner leaflet only accessible to AMPs by first translocating across the bilayer. However, as MST is a steady state measurement, it is unclear if this affects the observed values.

**Table 5:** Comparison of estimated vesicle and nanodisc sizes. *Surface area of both sides of the bilayer. **Assuming 100% DMPC composition with head area of 0.6 nm^2^ per lipid. ***Weight excludes SMA-QA polymer due to the uncertainty of the amount of SMA-QA per disc.

Model	Vesicle	SMA-QA nanodisc DMPC	SMA-QA nanodisc DMPC/PG
Radius (nm)	72	6	11
Surface area (nm^2^)*	120,000	260	790
Total number of lipids**	200,000	430	1320
Approx. weight**	140 MDa	290 kDa***	890 kDa***

Another difference between vesicles and nanodiscs, aside from the size, is the planarity of the lipid surfaces. Solubilised as LUVs, vesicles have a slightly convex surface curvature which introduces surface stress^49^. Nanodiscs on the other hand have a planar surface^50^—like the surface of the cell wall that is planar at a local level. In this context, nanodiscs may be a more representative model system. The difference in the curvature of the two systems may contribute to the difference in bindings observed towards the LUVs and SMA-QA nanodiscs. Peptides have demonstrated curvature-sensing properties, due to their preferential binding to packing defects that arise due to the curvature stress. Curvature-sensing is facilitated by hydrophobic motifs in peptides and can significantly improve the binding of peptides to more curved membranes^51–53^. The importance of hydrophobic motifs to peptides is also characterised by the correlation of the clustering of W residues with antimicrobial activity^4^. Indeed, such effects can be observed in the difference of measured K_D_s of **1**–**4** between the planar lipid conditions (SPR and SMA-QA) and curved lipid species (LUVs): the clustered-residue **1** and **3** having a tenfold improvement in binding to LUVs compared to the alternating-residue **2** and **4** which demonstrated only a fourfold improvement.

The difference in curvature between the two systems can affect the lipid phase in the bilayers of the nanodiscs and vesicles. The lipids solubilised as vesicles, have a uniform phase (at 25 °C this is near the *T*_*m*_ of DMPC and in the liquid-ordered phase)^54^. In contrast, the lipids in SMA-nanodiscs are less tightly packed than those solubilised as vesicles and have a reduced melting point^55^, and the same characteristics is expected of the SMA-QA nanodiscs. The central lipids of nanodiscs are in a more ordered phase^50^, while the outermost lipids, closest to the SMA-belt, are perturbed by the styrene groups of SMA^55^. AMPs are known to favour lipids that are in a more disordered phase and therefore one would expect heterogeneous interactions and distributions within the nanodiscs^56^.

**Figure 9:**
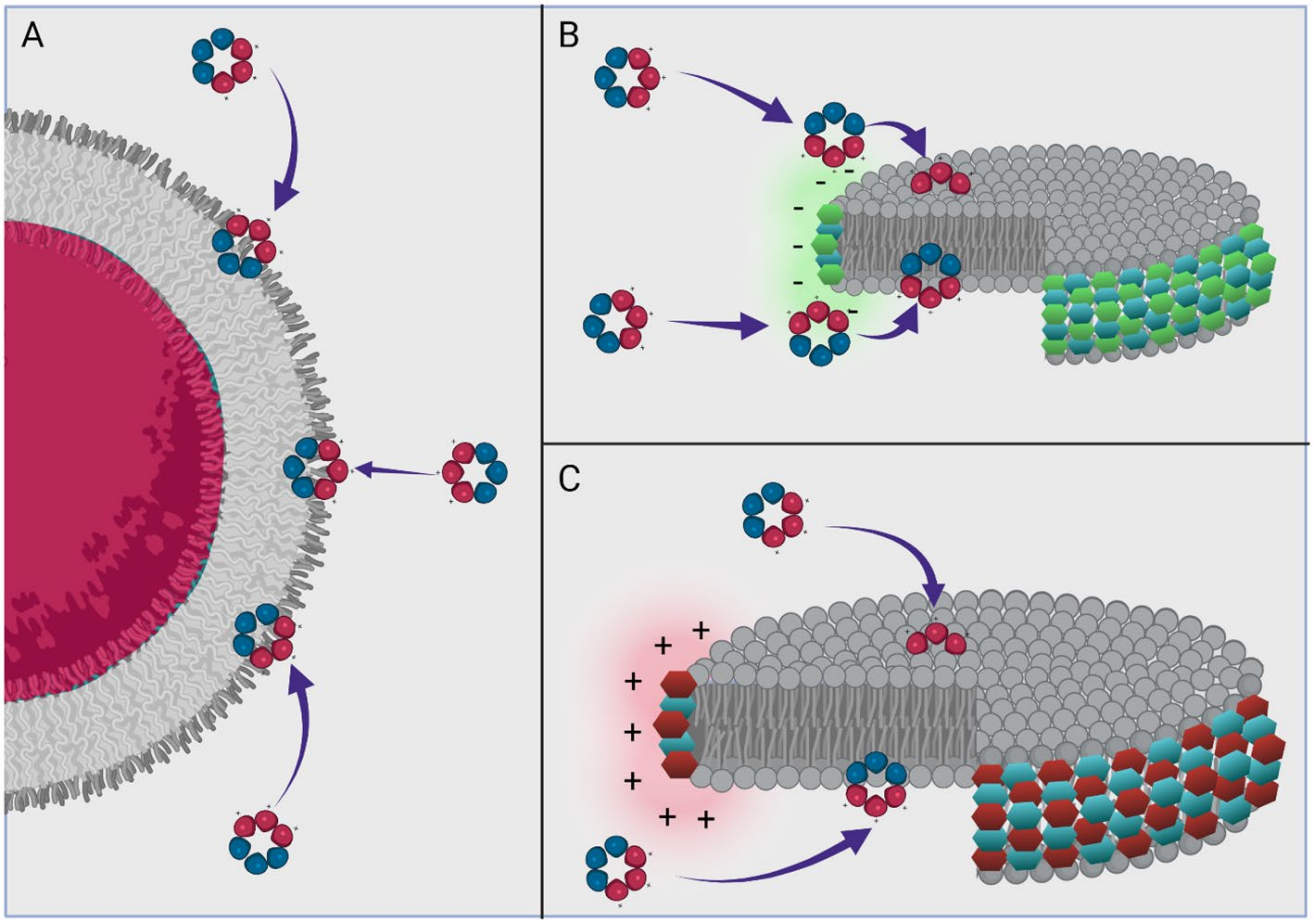
Visualisation of the difference in shape and characteristics of the lipid models and how this impacts the interactions of antimicrobial peptides.

In addition to the lipids, the role and impact of the respective belt polymers net charge is interesting to consider with regards to the two nanodisc systems. Some peptides have been shown to de-mix lipids into anionic lipid rich domains^57^. When such domains are formed, disorder at the domain boundaries can be exploited^58^. Polymer charge is known to affect the reconstitution of proteins and lipid species into nanodiscs^59,60^, and so it is therefore expected that it will influence the radial distribution of both the anionic DMPG and the studied cationic peptides. The negatively charged SMA will favourably interact with the cationic AMPs through electrostatic interactions, thereby also retaining the peptide in the proximity of the disordered lipid region. The unwanted effect of electrostatic interactions between SMA polymers and oppositely charged species has been previously reported by Ravula et al*.*^59^, where protein–nanodisc aggregates were reported due to strong electrostatic and hydrophobic interactions with the polymer. In MST, this was observed as a strong binding of the AMPs to both the SMA nanodiscs and the polymer alone, but any formation of nanodisc-aggregates was not apparent. In contrast, the cationic SMA-QA can have a repulsive effect on the AMPs, potentially repelling the AMPs from parts of the most disordered region near the polymer, containing the most favourable interactions on the nanodisc (Fig. 9). Similarly, Ravula et al*.*^59^ noted the beneficial effect of repulsive charges on the reconstitution of membrane proteins.

The cationic polymer charge could also, for example, have contributed to SMA-QA-DMPG forming larger and more heterogenous nanodiscs compared to the SMA-QA-DMPC and SMA nanodiscs (Table 5). The polymer charge may also have affected the final lipid content of the nanodiscs. ^31^P NMR estimated that the DMPG content in the SMA and SMA-QA nanodiscs were 2.7 and 4.5% respectively, from the initial vesicle content of 5% (Figs. S4–S7), demonstrating a negative effect on the yield for matched lipid and SMA charges.

The difference in the size of the two nanodisc preparations may also influence the interactions, with the larger SMA-QA PG containing discs having more lipids in an ordered phase than the smaller SMA discs. The role of the net charge of the belt is however expected to be the main driving factor behind the 20–200 times stronger interaction between the SMA nanodiscs and cationic AMPs compared to vesicles and SMA-QA nanodiscs. Surprisingly, while the respective *K*_*D*_ of the AMPs towards the two systems showed much more enhanced binding to the anionic SMA nanodiscs, *K*_*P*_ appeared to be measurable and consistent with SPR. Further studies are required to establish if the MST measured *K*_*P*_ reflects the true partitioning to the lipids in the SMA nanodiscs in this case, or if the apparent correlation is a product of cancellation effects. Accordingly, the interaction between the AMPs and the SMA-QA is approximately 4–10 times weaker than for the vesicles, likely affected by the difference in curvature and an electrostatic repulsion between the mutually cationic AMPs and SMA-QA. This could reduce the area of accessible lipids to interact with, in particular the lipids near the polymer that are in a less ordered phase.

The differences between the SPR and MST *K*_*D*_ could be explained by the experimental differences between the two methods. In MST, peptide response is monitored as a function of lipid concentration, whereas in SPR the added peptide mass to lipids immobilized on a chip is monitored as a function of peptide concentration. By keeping the AMP concentration fixed, any changes in activity due to concentration dependent processes, such as self-aggregation of AMPs, either pre- or post-binding, is not monitored. For the same reason, concentration dependent processes of the lipid system such as fusion, aggregation or turbidity are part of the response profile. Despite these differences, the vesicle MST and SPR produce binding data that are consistent relative to one another with regards to the ranking of the AMPs and the relative differences between the determined *K*_*D*_ values.

## Conclusions

We have demonstrated that MST, leveraging the intrinsic fluorescence of W, can be used to extract *K*_*D*_*,* and potentially *K*_*P*_, of W-rich AMPs towards model lipid bilayers in a fast and label-free manner. The measured *K*_*D*_ of **1**–**5** correlate well with both of their respective bactericidal activities (represented by MIC values) and with the ranking of binding obtained using SPR. We have successfully shown that MST can be used with various lipid particles (LUVs and nanodiscs) demonstrating robustness for studying membrane activities. The SMA-nanodiscs negatively charged polymer belt is not a suitable nanodisc scaffold for interaction studies with cationic AMPs, while SMA-QA nanodiscs are able to accurately reproduce the SPR derived results of the strongly binding AMPs. The results therefore highlight the need for careful consideration with regards to the lipid system to be used and the interaction to be analysed. Both SMA- and SMA-QA based nanodiscs are suitable constructs for ligands that do not directly interact with the polymers.

The extraction of the binding parameters of weak binders can be challenging due to potential interference from light scattering effects at high lipid concentrations compared to state-of-the-art SPR. On the other hand, MST analysis has much faster acquisition time and small sample/lipid requirements. With the introduction of automated hardware^61^, MST provides a low-cost and accessible alternative with high throughput for studying ligand-lipid interactions that is complementary to assays that detect AMP membrane disruptive properties such as for example WIND-PVPA, vesicle leakage, or patch-clamp experiments^62–64^.

MST would also be a highly suitable method to perform cost efficient scout experiments of sample conditions for lipid interactions, before initiating more advanced studies that rely on expensive sample preparation, labelling and/or instrument time.

## Methods

### Materials

Lipids were purchased from Avanti Polar Lipids via Sigma Aldrich (Merck KgaA, Darmstadt, Germany). MST consumables from Maricks AS (Oslo, Norway). SPR consumables were purchased from Cytiva Europe—Norge (Tyristrand, Norway). All other materials were purchased from Sigma Aldrich in analytical purity, unless otherwise stated. Peptides and SMA-QA were prepared in house.

The bacterial strains used were *E*. *coli* ATCC 25922 and *S*. *aureus* ATCC 9144. Overnight cultures and MIC assays were performed in cationic-adjusted BD BBL Mueller Hinton II Broth (MHB II, 212322, Becton, Dickson and Company, Sparks, MD, USA).

### Peptide synthesis

#### Linear peptide synthesis

2-chlorotrityl chloride resin (0.15 mmol, 1.0 meq, 150 mg) was swelled in DCM (5 mL) for 30 min. The resin was drained and treated with a solution of Fmoc-amino acid (0.3 mmol) and diisopropylethylamine (1.8 mmol, 313 mL) in DCM (5 mL). The resin mixture was left overnight under gentle agitation at room temperature. The resin mixture was drained, treated with MeOH (3 × 5 mL) to cap unreacted sites and dried with diethyl ether (3 × 5 mL). The linear peptides were prepared using an automated solid-phase peptide synthesizer (Biotage Initiator + Microwave System with Robot Sixty). The pre-loaded 2-chlorotrityl chloride resin was first swelled in DMF (20 min, 70 °C). Fmoc deprotections involved treatment of the resin with 20% piperidine/DMF (4.5 mL, 3 min) once at room temp. followed by a second treatment at 70 °C by microwave reactor. Amino acid couplings involved treatment of the resin with 4 eq. of Fmoc-amino acid (0.5 M in DMF), 4 eq. of HOBt (0.5 M in DMF), 4 eq. of HBTU (0.6 M in DMF) and 8 eq. of DIEA (2 M in NMP) for 5 min at 75 °C by microwave reactor for all Fmoc-amino acids except Fmoc-Arg(Pbf)-OH, which was coupled for 60 min at room temp. After each Fmoc deprotection and amino acid coupling, the resin was washed with DMF (4 × 4.5 mL × 45 s). After preparation of the resin-bound sidechain protected linear peptide, a final Fmoc deprotection and wash was preformed and the resin dried (3 × 5 mL MeOH, 3 × 5 mL Et_2_O). The resin-bound peptide was treated with 20% 1,1,1,3,3,3-Hexafluoro-2-propanol in DCM (2 × 5 mL × 15 min), followed by rinsing of the resin with DCM (5 mL). The filtrates were combined and concentrated under reduced pressure to yield the sidechain protected linear peptide.

#### Head-to-tail cyclisation and deprotection

The linear peptide (approx. 0.15 mmol) and diisopropylethylamine (0.9 mmol, 157 mL) were dissolved in DMF (10 mL) and added to a solution of PyBOP (0.45 mmol, 234 mg) in DMF (100 mL) under light stirring at room temperature. After 1–2 h (monitored for completion by mass spectrometry), the mixture was concentrated by reduced pressure and treated with a solution of TFA/triisopropylsilane/water (4 mL, 95%, 2.5%, 2.5%) then left to stand for 3 h. The mixture was concentrated under N_2_ gas flow followed by precipitation with ice-cold diethyl ether (15 mL). The precipitate was collected by filtration, washed with diethyl ether (15 mL), dissolved in 50% acetonitrile/water, and lyophilized to yield the crude, cyclic, sidechain deprotected peptide.

#### Purification

Peptides were purified by preparative reverse-phase HPLC (Waters 600 instrument with Waters 2487 Dual Absorbance detector) with a SunFire Prep. C18 OBD column (10 mm, 19 × 150 mm) using linear gradients of 0.1% TFA/water (buffer A) and 0.1% TFA/acetonitrile (buffer B) with a flow rate of 10 mL/min unless otherwise stated.

#### Analysis

Crude and final cyclic peptide products were analysed by FT-MS (Thermo Scientific LTQ Orbitrap XL instrument) and by analytical reverse-phase HPLC (Waters 2795 Alliance HT system with Waters 2996 PDA Detector), using an Ascentis C18 column (3 mm, 3 × 100 mm) and solvents of 0.1% TFA/water (buffer A) and 0.1% TFA/acetonitrile (buffer B) with a linear gradient of 0–60% buffer B over 15 min and a flow rate of 0.5 mL/min.

### Minimum inhibitory concentration (MIC) assay

The MICs for **1**–**5** were determined using the CLSI M07-A9 guidelines^65^. Working solutions were prepared in double distilled water containing max. 1% DMSO. A concentration range between 256–0.25 µg/mL was tested for each peptide. The bacterial inoculum was 1 × 10^6^ cells/mL and incubated 1:1 with each test compound in a polypropylene 96-well round-bottom plate (655209, Greiner Bio-One, Kresmmuenster, Austria). Each MIC test was performed in three biological replicates, consisting of four technical replicates. Positive controls (without antibiotics) and negative controls (without bacteria) were included for each technical replicate. The reference antibiotic erythromycin was included to assure quality control. The plates were incubated for 24 h at 37 °C. The MIC value was defined as the lowest concentration of compound resulting in no visible bacterial growth.

### Synthesis of SMA-QA

Following the procedure of Ravula et al.^33^ (2-aminoethyl)trimethylammonium chloride hydrochloride (9.38 mmol, 1.3 g) was added to a solution of styrene maleic acid anhydride (SMA, 1 g) in anhydrous DMF (5 mL), followed by trimethylamine (56.7 mmol, 5 mL) upon which the mixture took a dark yellow colour. The reaction mixture was stirred at 70 °C for 2 h, then cooled to room temperature, and precipitated with diethyl ether. The precipitate was washed 3 times with diethyl ether and dried *in vacuo*. The dried intermediate was dissolved in acetic anhydride (317 mmol, 30 mL), to which sodium acetate (8.05 mmol, 660 mg) and triethyl amine (1.98 mmol, 200 mg) were added. The reaction mixture was stirred at 80 °C for 12 h, cooled down, and precipitated in ether. The precipitate was washed 3 times in ether and dried *in vacuo*. The product was then dissolved in water and passed through a Sephadex LH-20 column. The product was collected and then lyophilized to give a crystalline brown powder and confirmed by IR stretching frequency shift from 1774 to 1693 cm^−1^ (Fig. S8).

### Vesicle preparation

DMPC and DMPC:5% DMPG vesicles were prepared by solubilising a known weight of lipid in chloroform with a small amount of methanol to assist in the dissolution of the PG lipid head groups. The chloroform stock was dried *in vacuo* to produce a lipid film, which was further dried for additional 3 h. The lipid film was solubilised in 10 mM TRIS buffer (pH 7.4) containing 100 mM NaCl to yield a 20 mM milky lipid stock.

To produce the working vesicle stock, 1 mL of vesicle stock was extruded 20 times through a 0.1 µm filter using an Avanti Lipids mini-extruder. Vesicle size was confirmed using a Malvern Zetasizer Nano ZS (Malvern Panalytical Ltd, Malvern, United Kingdom). 200 µL vesicle sample measured in 40 µL microcuvettes revealed vesicle diameters to be 144 ± 44 nm (DMPC) and 140 ± 48 nm (DMPC/PG).

### Nanodiscs preparation

The DMPC, and DMPC with 5% DMPG 21 mM vesicle stocks were used for the nanodisc preparation. The stocks were combined with an 8% SMA stock solution to yield a final SMA concentration of 1% for SMA nanodiscs. The stocks were combined with a 100 mg/mL SMA-QA stock to yield a final lipid:SMA-QA w/w ratio of 1:1.5. The combined SMA/SMA-QA and lipid mixture were incubated at room temperature overnight and purified by SEC. Fractions containing SMA/SMA-QA discs were concentrated using centrifugation filters. Total lipid concentration was determined by ^31^P NMR (Figs. S4–S7). Nanodisc size was confirmed using a Malvern Zetasizer Nano ZS (Malvern Panalytical Ltd, Malvern, United Kingdom). 200 µL nanodisc sample measured in 40 µL microcuvettes revealed nanodisc diameters to be 10.1 ± 3.0 nm (SMA DMPC), 9.2 ± 2.9 nm (SMA DMPC/PG), 12.8 ± 4.1 nm (SMA-QA DMPC), and 22.5 ± 11.8 nm (SMA-QA DMPC/PG) (Figs. S9–S11).

### SPR experimental procedure

The SPR measurements were performed using a T200 Biacore instrument (GE Healthcare, Oslo, Norway) at room temperature. An L1 chip was covered with extruded DMPC liposomes (1 mM in 10 mM HEPES buffer pH 7.4 with 100 mM NaCl) using a flowrate of 2 µL/min for 2400 s. Chip coverage was tested by injection of 0.1 mg/mL of bovine serum albumin for 1 min at 30 µL/min, with a change of < 400 RU indicating sufficient coverage.

An increasing concentration of tested peptides (peptides **1, 2, 3** and **4**-from 4 to 128 µM; peptide **5**—from 24 to 768 µM) was injected over immobilized vesicles with a flowrate of 15 µL/min for 200 s with a 400 s dissociation phase. The liposome surface was stabilized after each injection by three subsequent injections of 10 mM NaOH at 30 µL/min for 30 s each. Between experiments, the chip surface was cleaned by 20 mM CHAPS, 40 mM octyl-β-d-glucopyranoside and 30% ethanol in turn, with each solution injected for 1 min at 30 µL/min. The control flow cell was treated identically, with the exception that only the HEPES buffer solution was injected. The results were processed using in-house MATLAB scripts (MATLAB R2020a; scripts are available at https://github.com/MarJakubec).

### SPR data processing

*K*_*D*_ was obtained from a steady state analysis using the intensities at the 190 s dissociation time, using Eq. (1):^21^

1 $$R_{eq} = \frac{{cR_{\max } }}{{K_{D} + c}} + R_{off}$$

where *R*_eq_ is the response at the steady state equilibrium, c is the peptide concentration, *R*_max_ is the maximum response and *R*_*off*_ the response offset.

*K*_P_ was obtained from same steady state affinity values using the method presented by Figuera et al*.*, Eq. (2):^21^

2 $$\frac{{RU_{S} }}{{RU_{L} }} = \frac{{\gamma_{L} K_{P} \frac{{M_{S} }}{{M_{L} }}\left[ S \right]_{W} }}{{1 + \sigma \gamma_{L} K_{P} \left[ S \right]_{W} }}$$

where *RU*_*S*_ and *RU*_*L*_ are the relative responses of solute (peptides) and lipids respectively, *γ*_*L*_ is the molar volume of the lipids, *M*_*S*_ and *M*_*L*_ are the molecular mass of solute and lipid, respectively, and *[S]*_*W*_ is the concentration of solute in water. *K*_*P*_ and *σ* are obtained from fitting (with *σ* being lipid to solute ratio).

For *k*_*off*_ evaluation we have used the formalism of Figuera et al.^21^ for linearization of the dissociation process, where we have identified the contribution from two different populations in the dissociation response. *K*_*off*_ values were then obtained by Eq. (3) and averaged by Eq. (4):

3 $$S_{L} (t) = \alpha e^{{ - k_{{off,\alpha^{t} }} }} + \beta e^{{ - k_{{off,\beta^{t} }} }} + S_{L,r}$$

4 $$k_{off} = \frac{{\alpha k_{off,\alpha } + \beta k_{off,\beta } }}{\alpha + \beta }$$

where *S*_*L*_ is the linearized ratio of solute and lipid, *α* and *β* are individual populations, and *S*_*L,r*_ is the retained solute fraction.

### MST experimental procedure

All MST measurements were conducted on a NanoTemper Monolith NT. Labelfree instrument, using Monolith NT. Labelfree standard treated zero background capillaries.

A dilution series of vesicles/nanodiscs was prepared from 3 mM to 100 nM lipid concentrations, comprising 15 discrete samples, and an additional zero lipid sample, for a total of 16 lipid concentrations. Final MST samples were prepared as a combination of 25 µL lipid solution and 25 µL 5 µM peptide solution (Table S1 in the Supporting Information).

MST measurements were conducted with excitation power set to 15%, with the MST power set to high. Laser time settings were: 3 s pre-laser, 30 s on time, and 3 s after heating. *F*_*Hot*_ was taken from the T-jump period after 1.5 s and *F*_*Cold*_ taken in the second prior to IR laser activation. For the evaluation of *K*_*P*_, the initial fluorescence was taken as the value reported during the period before the application of the laser. The MST response and initial fluorescence were extracted directly as a text file for further processing in MATLAB.

### MST data processing

The dissociation constant *K*_*D*_ describes the equilibrium between the concentration of bound and unbound ligand^40^.

5 $$K_{D} = \frac{{\left[ {AMP} \right]\left[ {Lipid} \right]}}{{\left[ {AMPLipid} \right]}}$$

In a typical binding experiment that yields a sigmoidal curve, the Hill equation can be fitted to yield *K*_*D*_:^66^

6 $$y = y_{0} + \frac{{E_{Max} \left[ {Lipid} \right]^{n} }}{{K_{D}^{n} + \left[ {Lipid} \right]^{n} }}$$

where y is the MST response, *y*_0_ is the MST response of the AMP in an aqueous solution (i.e., in the absence of lipids), *n* is the Hill coefficient, and *E*_*Max*_ is the maximal effect of the tested substrate^66^. The removal of outlying MST response points was necessary. Erroneous points were identified by poor MST trace shapes or higher than expected initial fluorescence that was absent in the other replicates or subsequent points, but otherwise no further treatment of data was necessary. In all instances the MST response was plotted against log lipid concentration in nM and fit to Eq. (6).

The partition coefficient *K*_*p*_ defines the preference of a solute for an aqueous or lipidic environment, with a larger *K*_*p*_ indicating a greater preference for the lipidic environment.

7 $$K_{P} = \frac{{S\left[ {Lipid} \right]}}{{S\left[ {Aqueous} \right]}}$$

The *K*_*p*_ of a molecule can be determined experimentally by observing changes in fluorescent intensity in the presence of an increasing concentration of lipid, and fitting to Eq. (8)^43^.

8 $$\frac{I}{{I_{aq} }} = 1 + \frac{{\left( {K_{p} V_{m} \left[ {Lipid} \right]\frac{{I_{L} }}{{I_{aq} }}} \right)}}{{1 + \left( {K_{P} V_{m} \left[ {Lipid} \right]} \right)}}$$

In Eq. (8) the fluorescence intensity of the AMP (I) is normalised to the fluorescence intensity of the AMP in an aqueous environment (*I*_*aq*_), *V*_*m*_ is the molar volume of the lipids and *I*_*L*_ is the fluorescence intensity of the AMP in the lipidic environment. For *V*_*m*_, the average molar volume of the lipid composition is used. In the case of the DMPC only environments it is taken as the *V*_*m*_ of DMPC (1.023 nm^3^), and in the DMPC-DMPG mixture it is the weighted average relative to the composition used (*V*_*m DMPG*_ = 0.997 nm^3^)^54^.

### Supplementary Information

Supplementary Information.

## Data Availability

The datasets generated and analysed during the current study are available in the UiT Open Research Data repository 10.18710/XZB5KI.
