# Supplementary material for: Label-free measurement of antimicrobial peptide interactions with lipid vesicles and nanodiscs using microscale thermophoresis
Source: Sci Rep. 2023 Aug 3;13:12619. doi: 10.1038/s41598-023-39785-0 (PMC10400562; doi:10.1038/s41598-023-39785-0)
Supplement: Supplementary file 1 — Supplementary Information. [file 41598_2023_39785_MOESM1_ESM.docx]

**Supplemental materials: Label-free measurement of antimicrobial peptide interactions with lipid vesicles and nanodiscs using microscale thermophoresis**

Philip Rainsford,^a^ Fredrik G. Rylandsholm, ^a^ Martin Jakubec,^a^ Mitchell Silk,^a^ Eric Juskewitz,^b^ Johanna U. Ericson,^b^ John-Sigurd Svendsen,^a^ Richard A. Engh,^a^ Johan Isaksson^*,a,c^

^a^ Department of Chemistry, Faculty of Science and Technology, UiT the Arctic University of Norway, 9019 Tromsø, Norway

^b^ Research Group for Host Microbe Interactions, Department of Medical Biology, Faculty of Health Sciences, UiT the Arctic University of Norway, 9019 Tromsø, Norway

^c^ Natural products and Medicinal Chemistry, Department of Pharmacy, Faculty of Health Sciences, UiT the Arctic University of Norway, 9037 Tromsø, Norway

*johan.isaksson@uit.no

**Supp. 1**

**Cyclic Peptide Purification and characterisation:**

**1**; *Cyclo*(WWWRRR). *Linear precursor:* H_2_N-Arg(Pbf)-Arg(Pbf)-Arg(Pbf)-Trp(Boc)-Trp(Boc)-Trp(Boc)-OH. *Cyclic: cyclo*(Trp-Trp-Trp-Arg-Arg-Arg). Purification gradient: 15-75% buffer B over 60 min, t_R_ = 18 min (33% buffer B). Yield: 46.7 mg (30.0% relative to linear precursor) as a white solid. ESI-FTMS [M + H]^+^ calculated: 1027.5419, found: 1027.5473, [M + 2H]^2+^ calculated: 514.2785, found: 514.2770, [M + 3H]^3+^ calculated: 343.1882, found: 343.1872.

**2**; *Cyclo*(WRWRWR). *Linear precursor:* H2N-Arg(Pbf)-Trp(Boc)-Arg(Pbf)-Trp(Boc)-Arg(Pbf)-Trp(Boc)-OH. *Cyclic: cyclo*(Trp-Arg-Trp-Arg-Trp-Arg). Purification gradient: 5-65% buffer B over 60 min, tR = 25 min (30% buffer B). Yield: 26.9 mg (22.6% relative to linear precursor) as a white solid. ESI-FTMS [M + 2H]2+ calculated: 514.2785, found: 514.2779, [M + 3H]3+ calculated: 343.1882, found: 343.1883.

**3**; *Cyclo*(WWWKKK). *Linear precursor:* H2N-Lys(Boc)-Lys(Boc)-Lys(Boc)-Trp(Boc)-Trp(Boc)-Trp(Boc)-OH. *Cyclic: cyclo*(Trp-Trp-Trp-Lys-Lys-Lys). Purification gradient: 10-70% buffer B over 60 min, tR = 22 min (32% buffer B). Yield: 49.5 mg (39.0% relative to linear precursor) as a white solid. ESI-FTMS [M + H]+ calculated: 943.5307, found: 943.5354, [M + Na]+ calculated: 965.5126, found: 965.5159, [M + 2H]2+ calculated: 472.2693, found: 472.2702.

**4**; *Cyclo*(WKWKWK). *Linear precursor:* H2N-Trp(Boc)-Lys(Boc)-Trp(Boc)-Lys(Boc)-Trp(Boc)-Lys(Boc)-OH. *Cyclic: cyclo*(Trp-Lys-Trp-Lys-Trp-Lys). Purification gradient: 5-65% buffer B over 60 min, tR = 21 min (26% buffer B). Yield: 40.7 mg (41.8% relative to linear precursor) as a white solid. ESI-FTMS [M + H]+ calculated: 943.5307, found: 943.5308, calculated: 472.2693, found: 472.2689.

**5**; *Cyclo*(LWwNKr). *Linear precursor:* H_2_N-Trp(Boc)-D-Trp(Boc)-Asn(Trt)-Lys(Boc)-D-Arg(Pbf)-Leu-OH. *Cyclic: cyclo*(Leu-Trp-D-Trp-Asn-Lys-D-Arg). Purification gradient: 10-70% buffer B over 60 min, 6 mL/min, t_R_ = 23 min (33% buffer B). Yield: 35.1 mg (44.9% relative to linear precursor) as a white solid. ESI-FTMS [M + H]^+^ calculated: 884.4895, found: 884.4895, [M + 2H]^2+^ calculated: 442.7487, found: 442.7483.

**Table** S**1**: Table of lipid and peptide concentrations for MST

| MST Sample | Final lipid concentration (nM) | Final AMP concentration (nM) |
| --- | --- | --- |
| 1 | 1500000 | 2500 |
| 2 | 1250000 | 2500 |
| 3 | 500000 | 2500 |
| 4 | 250000 | 2500 |
| 5 | 125000 | 2500 |
| 6 | 50000 | 2500 |
| 7 | 25000 | 2500 |
| 8 | 12500 | 2500 |
| 9 | 5000 | 2500 |
| 10 | 2500 | 2500 |
| 11 | 1250 | 2500 |
| 12 | 500 | 2500 |
| 13 | 250 | 2500 |
| 14 | 125 | 2500 |
| 15 | 50 | 2500 |
| 16 | 0 | 2500 |

**Figure** S**1**: MST traces of increasing DMPC/PG vesicle concentration with **1** (red) and with no peptide (green), demonstrating the typical differences of the MST traces when peptide is present. Notably a more irregular trace can only be observed in the absence of the fluorophore carrying peptides, with a smaller thermophoretic change.


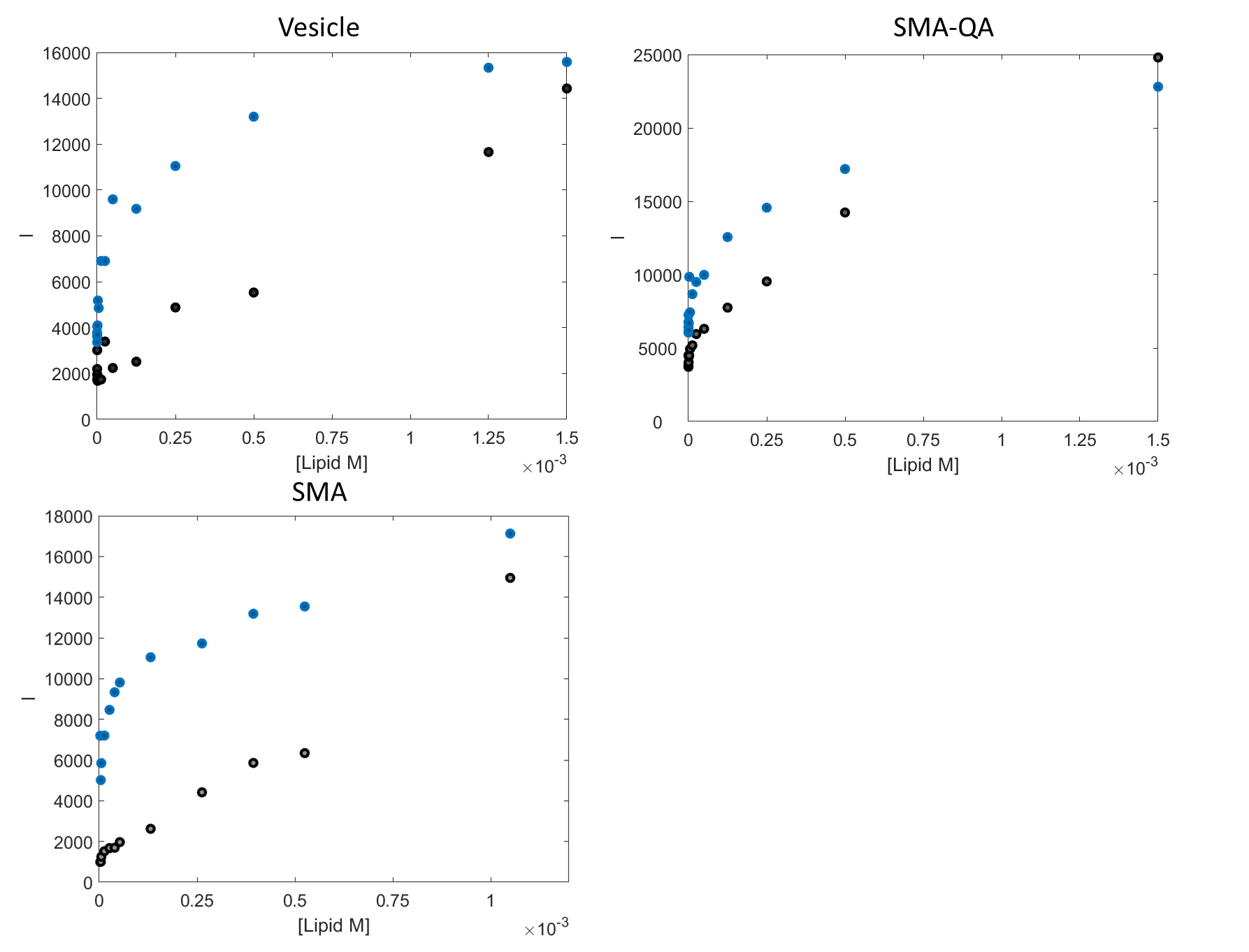


**Figure** S**2**: Fluorescent intensities of samples in the presence of 1 (blue) and without any peptide i.e. lipid only (gray). Both SMA and vesicle samples show markedly higher intensities when an AMP is present in the samples with a (generally) parabolic line shape. In SMA-QA the lipid only sample has a higher fluorescent intensity compared to SMA and vesicles, however the parabolic line shape is clearly differentiated when an AMP is present.


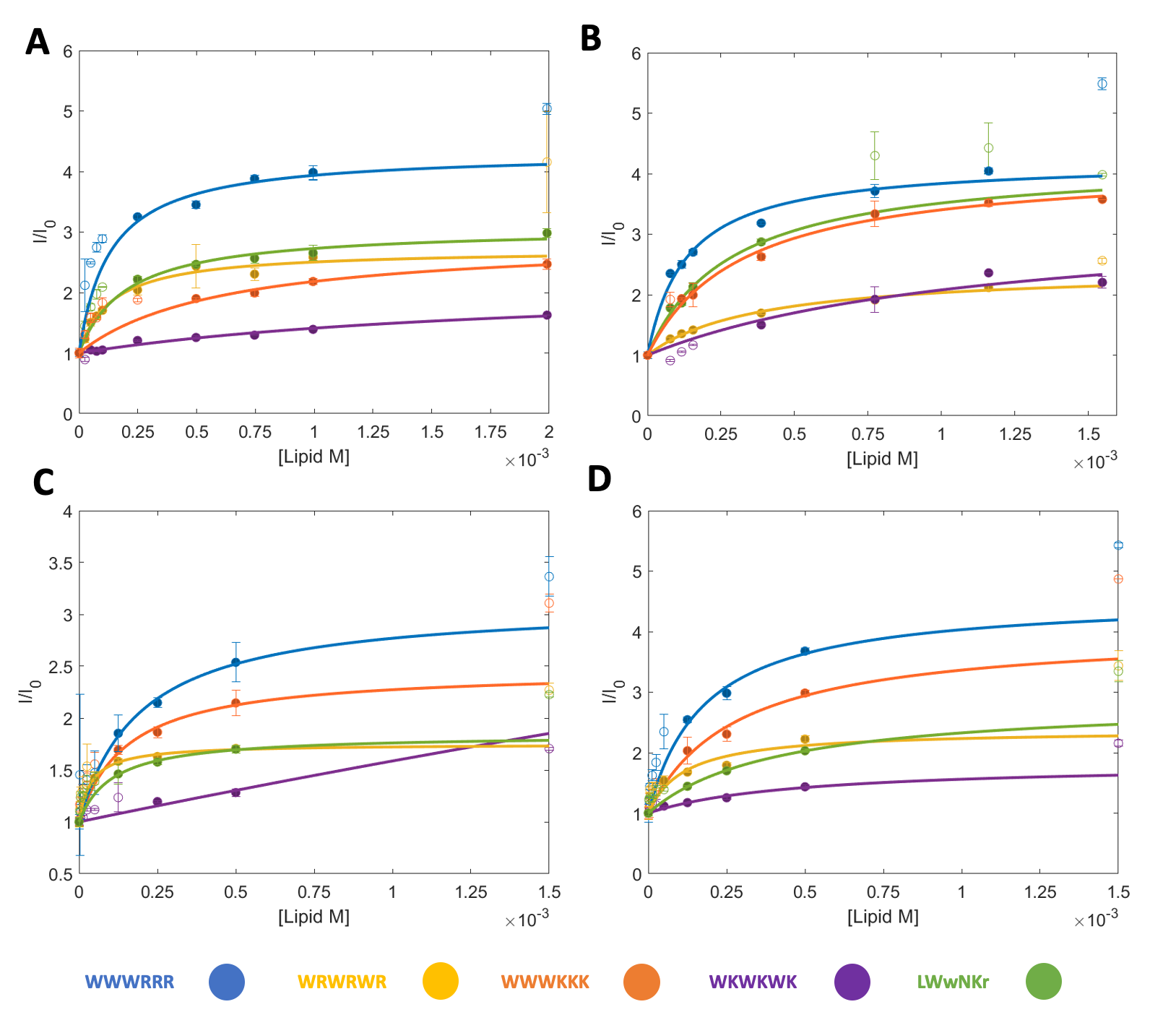


**Figure** S**3**: Initial fluorescence and K_P_ fits of 1-5 in different lipid compositions of nanodiscs. **A**: SMA 100% DMPC. **B**: SMA 95% DMPC with 5% DMPG. **C**: SMA-QA 100% DMPC. **D**: SMA-QA 95% DMPC with 5% DMPG. Lines and filled circles indicate the fit and the points used, unfilled circles are points not included. Error bars represent the range in the triplicates.


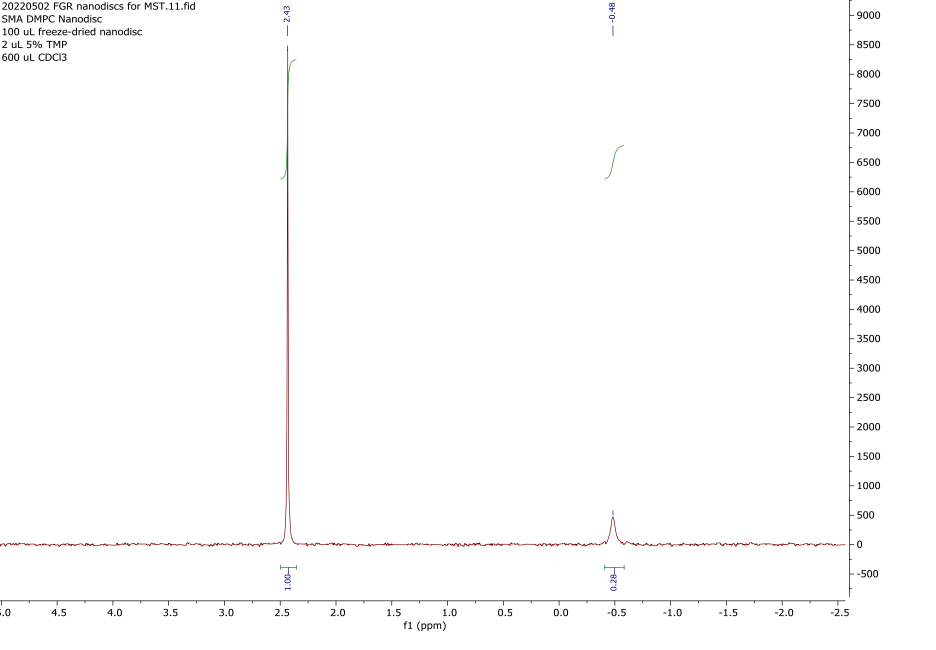


**Figure S4:** *^31^P NMR spectrum of freeze dried SMA DMPC nanodiscs dissolved in CDCl_3_ with TMP internal standard. From the integrals and volumes, we calculate the DMPC concentration to be 2.39 mM.*


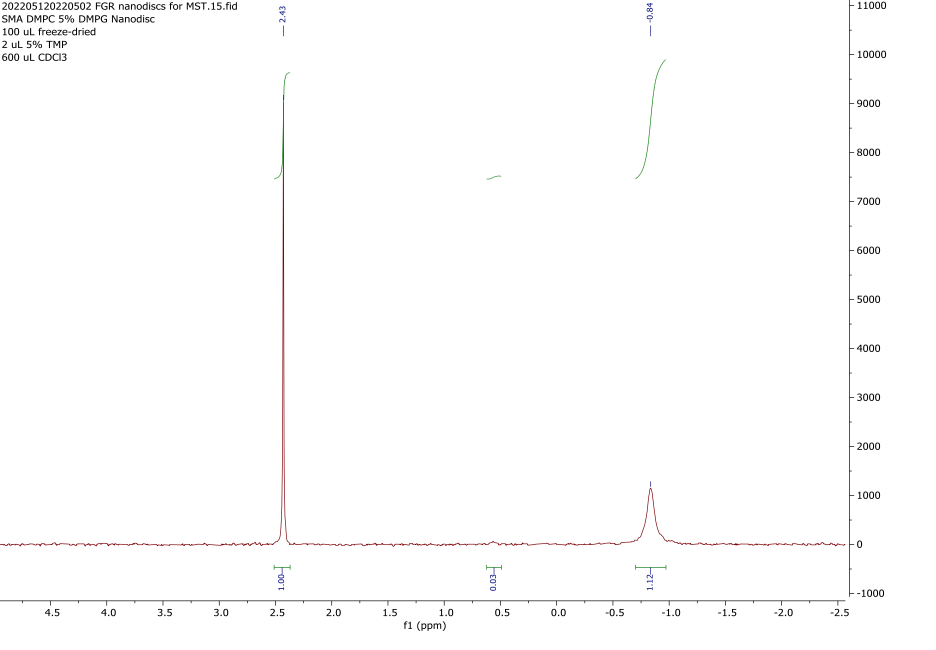


**Figure S5***: ^31^P NMR spectrum of freeze dried SMA DMPC 5% DMPG nanodiscs dissolved in CDCl_3_ with TMP internal standard. From the integrals and volumes, we calculate the DMPC concentration to be 9.57 mM and the DMPG concentration to be 0.26 mM (2.72% DMPG).*


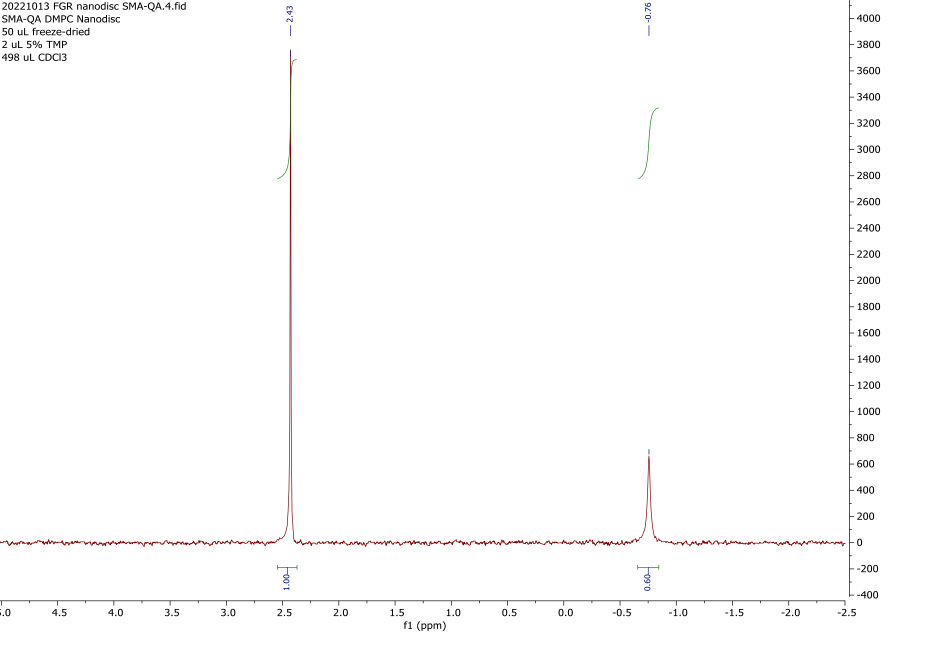


**Figure S6:** *^31^P NMR spectrum of freeze dried SMA-QA DMPC nanodiscs dissolved in CDCl_3_ with TMP internal standard. From the integrals and volumes, we calculate the DMPC concentration to be 10.26 mM.*


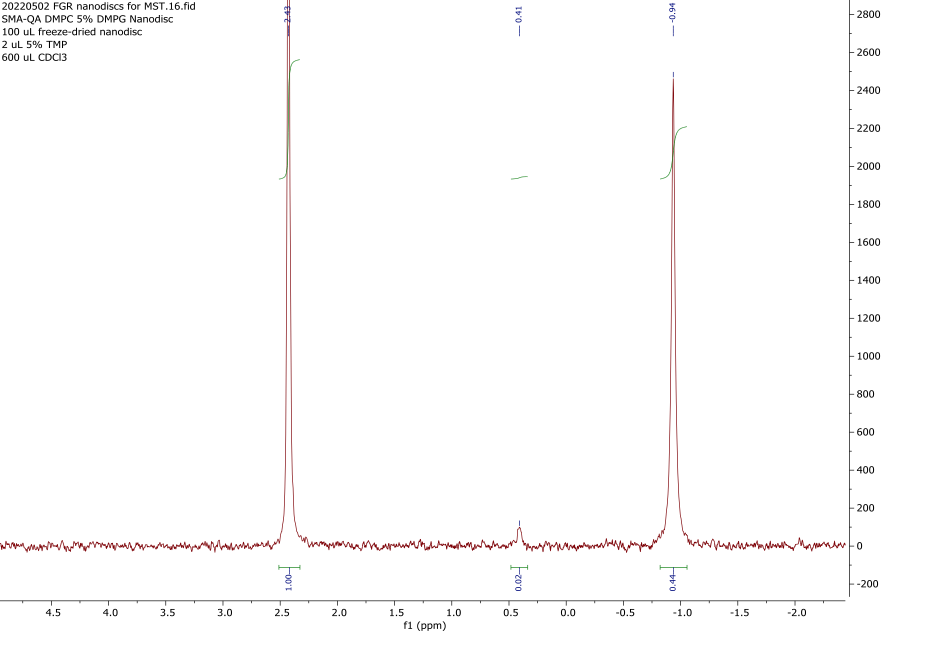


**Figure S7:** *^31^P NMR spectrum of freeze dried SMA-QA DMPC 5% DMPG nanodiscs dissolved in CDCl_3_ with TMP internal standard. From the integrals and volumes, we calculate the DMPC concentration to be 5.13 mM and the DMPG concentration to be 0.23 mM (4.5% DMPG).*


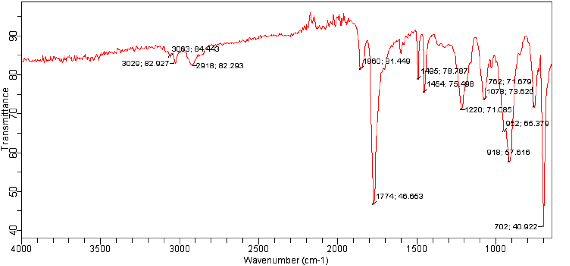


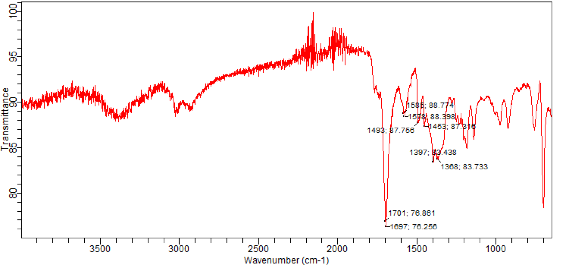


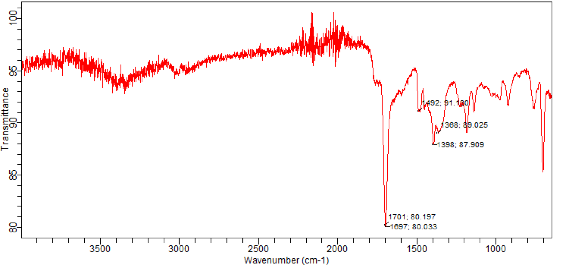


**Figure S8**: *TOP: FT-IR spectrum of SMA anhydride, the starting material for the synthesis of SMA-QA. MIDDLE: FT-IR spectrum of SMA-QA, synthesized from SMA anhydride. BOTTOM: FT-IR spectrum of SMA-QA, bought from BioNordika AS, and produced by anatrace.*

*
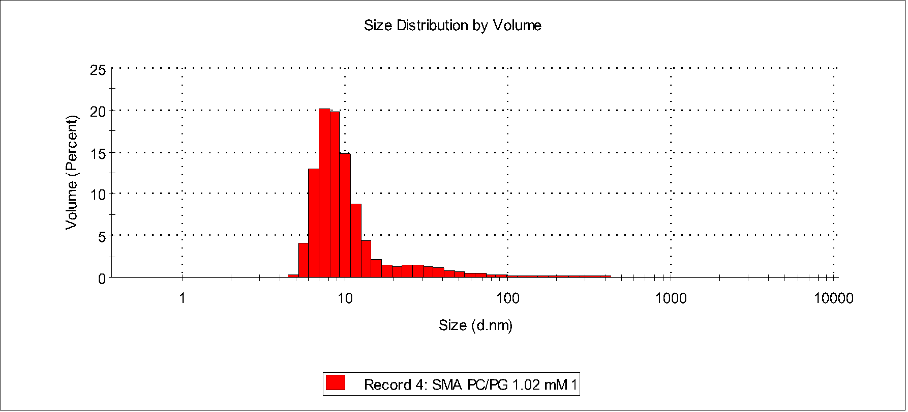
*

**Figure S9:** *DLS Size distribution by volume of SMA DMPC/PG nanodiscs.*

*
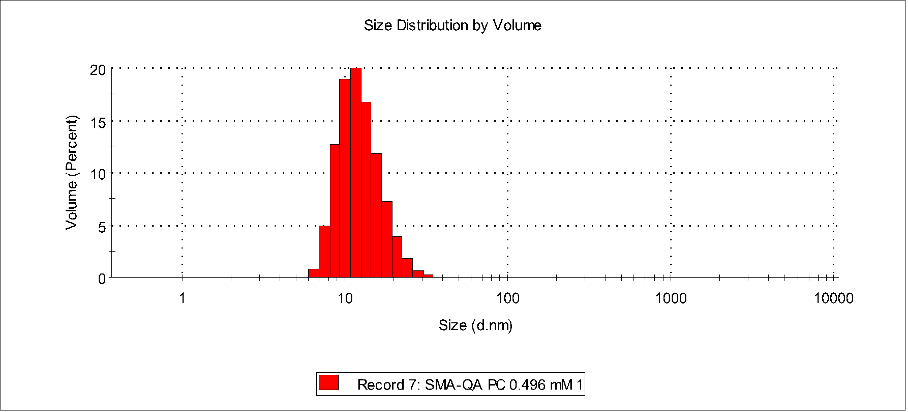
*

**Figure S10***: DLS Size distribution by volume of SMA-QA DMPC nanodiscs.*

*
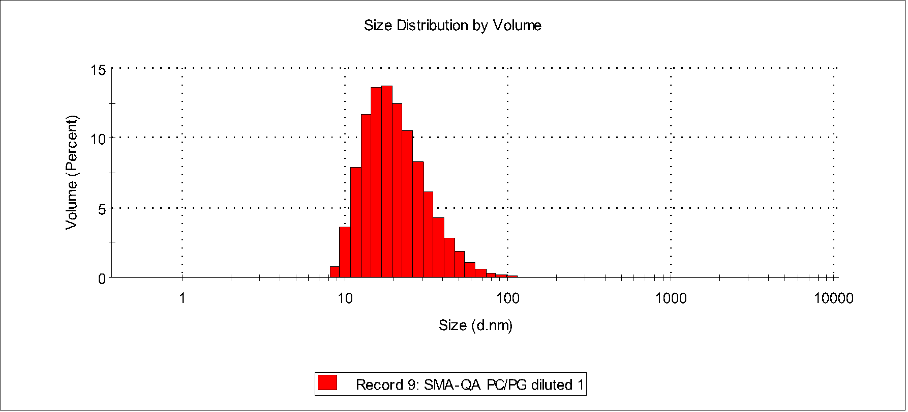
*

**Figure S11:** *DLS Size distribution by volume of SMA-QA DMPC/PG nanodiscs.*
